# Characterization of cancer omics and drug perturbations in panels of lung cancer cells

**DOI:** 10.1038/s41598-019-55692-9

**Published:** 2019-12-20

**Authors:** Ayako Suzuki, Keiichi Onodera, Ken Matsui, Masahide Seki, Hiroyasu Esumi, Tomoyoshi Soga, Sumio Sugano, Takashi Kohno, Yutaka Suzuki, Katsuya Tsuchihara

**Affiliations:** 1 Department of Computational Biology and Medical Sciences, Graduate School of Frontier Sciences, The University of Tokyo, Chiba, Japan; 2 Division of Translational Informatics, Exploratory Oncology Research and Clinical Trial Center, National Cancer Center, Chiba, Japan; 3 Bio Science & Engineering Laboratory, Fujifilm Corporation, Kanagawa, Japan; 4 Research Institute for Biomedical Sciences, Tokyo University of Science, Chiba, Japan; 5 Institute for Advanced Biosciences, Keio University, Yamagata, Japan; 6 Division of Genome Biology, National Cancer Center Research Institute, Tokyo, Japan

**Keywords:** Epigenomics, Transcriptomics, Cancer genomics, Target identification

## Abstract

To better understand the disruptions of transcriptional regulations and gene expression in lung cancers, we constructed a multi-omics catalogue of the responses of lung cancer cells to a series of chemical compounds. We generated and analyzed 3,240 RNA-seq and 3,393 ATAC-seq libraries obtained from 23 cell lines treated with 95 well-annotated compounds. To demonstrate the power of the created multi-omics resource, we attempted to identify drugs that could induce the designated changes alone or in combination. The basal multi-omics information was first integrated into co-expression modules. Among these modules, we identified a stress response module that may be a promising drug intervention target, as new combinations of compounds that could be used to regulate this module and the consequent phenotypic appearance of cancer cells have been identified. We believe that the multi-omics profiles generated in this study and the strategy used to stratify them will lead to more rational and efficient development of anticancer drugs.

## Introduction

Cancer clinical sequencing analyses at a varying scales identify the genotypes of cancers, including the driver mutations in lung adenocarcinoma cells^1,2^. These mutations are subjected to further analysis as potential targets for molecular targeting drugs. For example, EGFR tyrosine kinase inhibitors (e.g., gefitinib and erlotinib) and ALK inhibitors (e.g., crizotinib) are administered to patients with *EGFR* mutations and *ALK* fusions, respectively^3–5^. Suitable therapeutic approaches based on the genomic mutation patterns of each cancer have been successful. Nevertheless, approximately 30% of patients with lung adenocarcinoma do not benefit from this approach because appropriate molecular targeting drugs are not available^6^.

To alleviate shortages in the repertoire of chemical compounds that may serve as “seeds” for eventual drug development, current approaches utilize “high-throughput screening” which is becoming more expensive. Even when starting with millions of compounds, eventual success in obtaining a desirable drug cannot always be guaranteed. To overcome this drawback, the intensive utilization of the accumulated knowledge of genomes, epigenomes, and the transcriptional activities of cells and their molecular networks is currently underway; for example, the Encyclopedia of DNA Elements (ENCODE) database has been used for such studies^7^.

Indeed, omics information for cell lines, which are frequently used for cancer research, has been rapidly accumulated^8–10^. By making use of panels of cancer cell lines, numerous studies have revealed the details of the associations among genotypes and phenotypes. For example, the Cancer Cell Line Encyclopedia (CCLE) and other large projects have generated data on the genotypes and cellular responses to pharmacological intervention of hundreds of cancer cell lines^10–12^. Furthermore, target screening systems were applied to enrich the informational resources for the panels, particularly when novel genetic vulnerabilities in the cancer cells were investigated^13–16^. These high-throughput screens provide useful information about how the products of individual genes or their combinations could efficiently kill cancer cells and could therefore be targeted by drugs. Moreover, the Connectivity Map (CMAP)^17^ project compiled a large collection of gene expression profiles of cell lines treated with drugs, which predicted the modes of actions of drugs through comparisons of the similarities among transcriptional profiles^18^. In particular, large L1000 CMAP datasets comprised more than one million profiles^19^.

Nevertheless, in these large-scale drug screening projects, the systematic integration of different layers of omics information, including epigenome status and perturbations, even when epigenome targeting drugs have been considered, have not been fully implemented. Indeed, the highly diverse phenotypes of cancer cells, which vary according to the originating patient characteristics and tumor stages, may be mediated by the diverse activities of transcriptomes and epigenomes^20^. Therefore, without understanding these variations, it may be impossible to develop effective anti-cancer drugs.

This study attempted to construct a basic resource for multi-omics information about cell lines and their drug perturbations. We first determined the multi-omics features of representative lung adenocarcinoma cell lines^8,21,22^. The resulting dataset, which we generated using whole genome sequencing, comprised the representative patterns of the driver mutations in lung adenocarcinoma, such as *EGFR* and *KRAS* point mutations, *ALK* and *RET* fusions, and the amplifications of genes such as *ERRB2* and *MET* genes^1,2,23,24^. Genomic mutational information was further associated with epigenomic and transcriptomic patterns, which were measured using ChIP-seq analysis of eight chromatin marks, bisulfite sequencing of DNA methylation and RNA-seq and TSS-seq analysis^25,26^.

The catalogue was further expanded to include information about how the transcriptomic and epigenomic activities of cells were perturbed in response to small compounds. To enhance the data output, we developed a new system to facilitate RNA-seq and ATAC-seq.^27^. To extract biological interpretations from the substantial amount of created data, we further integrated the datasets of the transcriptional regulatory signatures, consisting of the genomic and metabolomic signatures and their drug-perturbation patterns. Here, by systematically exploiting in-depth multilayered cellular omics information, we generated a resource for the development of more rational and efficient drug development strategies.

## Results and Discussion

### Overview of the study design

**Figure 1:**
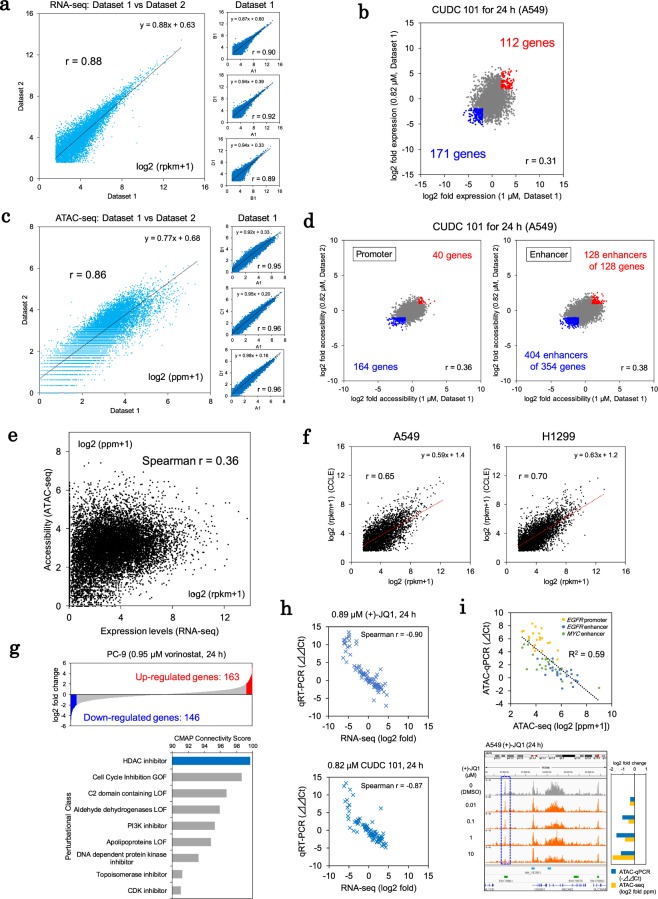
High-throughput transcriptome and epigenome perturbations. (**a**) Reproducibility of the RNA-seq data. The gene expression levels of the A549 cells (DMSO control) were compared. Left panel: the reproducibility among the different plates was evaluated. Right panel: the datasets within the same plates were compared. The Pearson correlation coefficients are shown in the inset. (**b**) Expression changes in A549 cells treated with CUDC 101. The fold changes in expression were calculated and compared between dataset 1 and dataset 2 for similar treatment conditions (≥4-fold and ≤1/4-fold changes are shown in red and blue, respectively). The Pearson correlation coefficients are shown in the inset. (**c**) Reproducibility of the ATAC-seq data. The signal intensities reflecting the open chromatin status are shown for the promoter regions of genes in the A549 cells in a manner similar to that shown in (**a**). (**d**) Quantification of the changes in the open chromatin status in A549 cells treated with CUDC 101. The fold changes in the intensities of the ATAC-seq signals were compared between the datasets in a manner similar to that shown in (**b**) (≥2 and ≤1/2-fold changes are shown in red and blue, respectively). (**e**) Association between the expression levels (RNA-seq) and chromatin accessibility of the promoter regions (ATAC-seq). (**f**) Comparison of transcriptome profiles between the in-house, high-throughput RNA-seq and public CCLE RNA-seq data. Two examples from among the 15 cell lines are shown. The Pearson correlation coefficients are shown in the inset. (**g**) Comparison of the transcriptome mode of action according to the CMAP signatures. The up/downregulated genes in PC-9 cells treated with vorinostat were determined as part of the signature of vorinostat (genes with ≥4 and ≤1/4-fold changes under vorinostat treatment, upper panel). The connectivity scores of the vorinostat signatures are shown for each CMAP perturbational class (lower panel). (**h**) qPCR validation of the RNA-seq data (see Supplementary Methods). The Spearman correlation coefficients are shown in the inset. (**i**) qPCR validation analysis of the ATAC-seq data (see Supplementary Methods). The intensities in terms of the open chromatin status were validated for three regulatory regions (upper panel). A549 cell ATAC-seq tags obtained during treatment with (+)-JQ1 were represented in the Integrative Genomics Viewer (IGV) (autoscale) (lower panel). The graph indicates the fold changes in the intensities of the ATAC-seq peaks (blue dashed box) (lower right).

**Figure 2:**
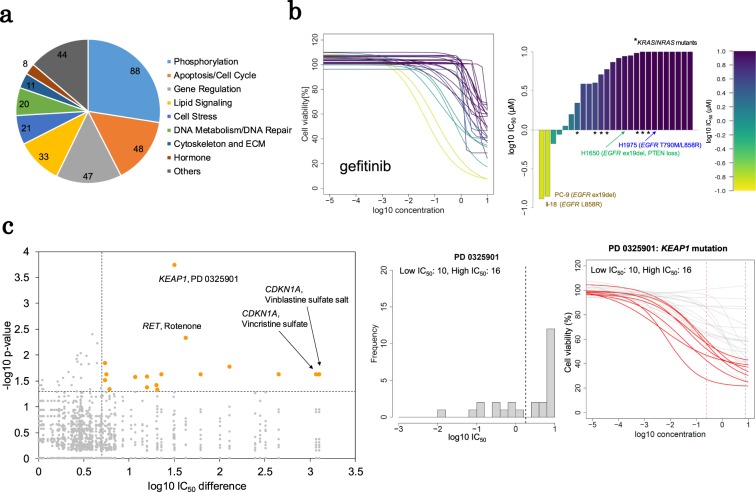
Molecular profiles predicted the drug sensitivities. (**a**) The classifications of 319 compounds tested for their effects on cell viability. (**b**) Differences in the sensitivity to gefitinib. The 8-point dose-response curve of gefitinib is shown (left). The log10 IC_50_ values of gefitinib are also shown in the bar graph (right). Each bar represents a cell line. The y axis indicates the IC_50_ values (µM) with log10 transformation. The mutational status of *EGFR*, *KRAS* and *NRAS* are shown in the inset. The colors are graded from yellow to purple depending on the sensitivity, and the color bar is shown in the margin. (**c**) Association between the mutations and drug sensitivity. In the left plot, the associated gene-compound pairs are shown. Orange dots represent significant pairs. The distribution of the IC_50_ values of PD 0325901 is shown in the middle graph. According to the IC_50_ values, the cell lines were divided into two groups (dashed line). The dose-response curve of PD 0325901 is shown in the right panel. The curves representing the KEAP1 mutant cells are red.

**Figure 3:**
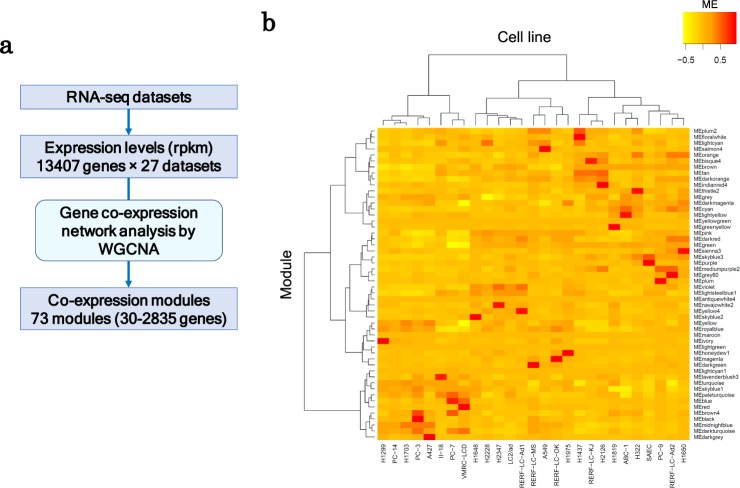
Transcriptional trends of lung adenocarcinoma based on gene co-expression networks. (**a**) A simple workflow of the WGCNA analysis. (**b**) The heat map of the module activities in the lung cancer cell lines. The eigengenes of the modules are represented as the module activities. The hierarchical clustering was conducted using Ward’s method with Pearson correlation. For the selected modules, the clustering analysis is provided in Fig. 6a.

**Figure 4:**
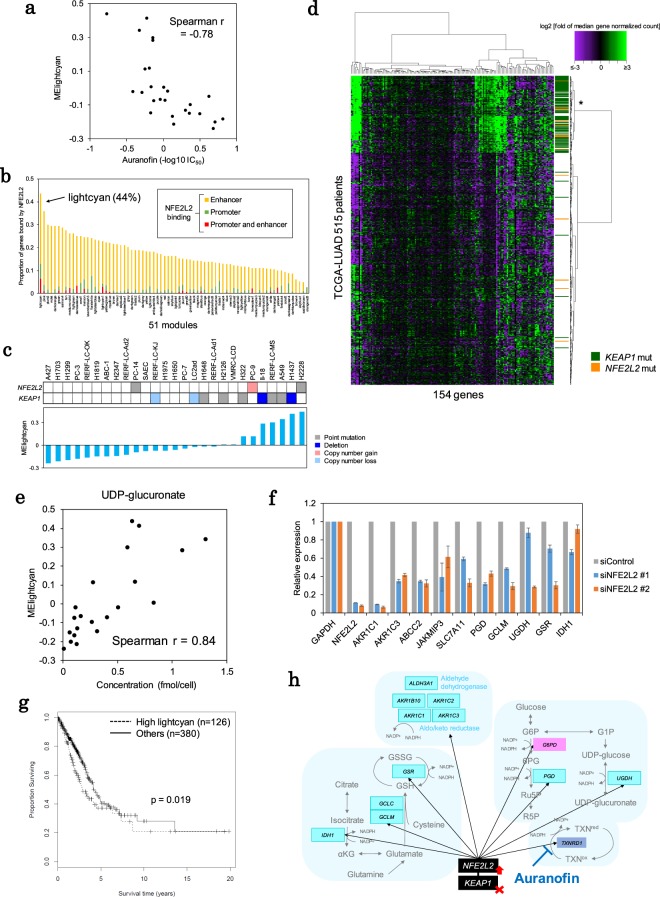
Antioxidative stress modules. (**a**) Sensitivity to auranofin was associated with activity of the “lightcyan (putative redox)” module. The IC_50_ values were compared with the module eigengenes of the module. The Spearman correlation coefficient is shown in the inset. (**b**) NFE2L2 targets were enriched within the “lightcyan (putative redox)” module members. The proportion of genes with NFE2L2 binding sites in the promoter or enhancer is shown for each module. (**c**) The association between *KEAP1*-*NFE2L2* mutations and “lightcyan (putative redox)” module activity. The mutational patterns of *KEAP1* and *NFE2L2* are shown in the upper panel. The module activity is shown in the lower panel. The cell lines are ordered according to the ME for the module. (**d**) Expression patterns of the “lightcyan (putative redox)” module members in 515 TCGA-LUAD cases. The median fold changes in the expression levels are shown in the heat map. The mutational statuses of the *KEAP1* and *NFE2L2* genes are shown in the side bar in green and orange, respectively. The color key is shown in the margin. (**e**) Correlation between the amounts of UDP-glucuronate and “lightcyan (putative redox)” module activity. The Spearman correlation coefficient is shown in the inset. (**f**) *NFE2L2* knockdown downregulated “lightcyan (putative redox)” module members. (**g**) Kaplan-Meier analysis of TCGA-LUAD cases based on the activity of the “lightcyan (putative redox)” module. Overall survival time was compared between cases with high module activity (asterisk in **d**) and other cases. (**h**) The putative redox module. Representative member genes in the “lightcyan (putative redox)” module are shown along the associated metabolic pathways.

**Figure 5:**
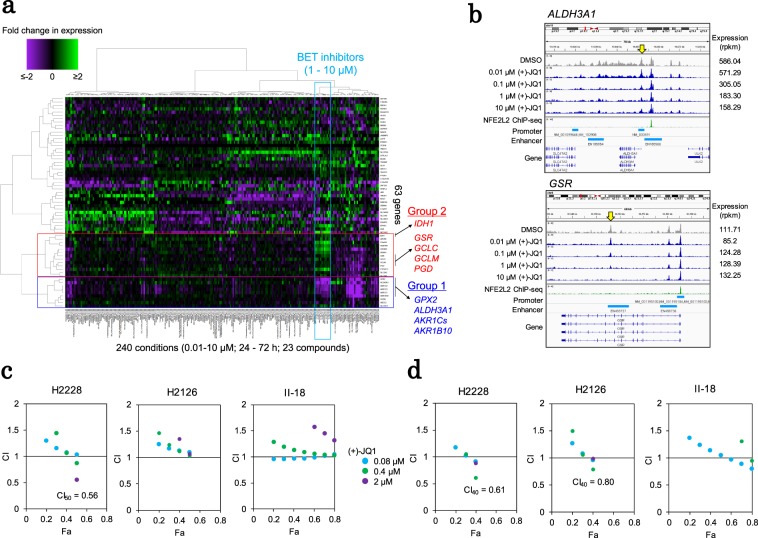
Epigenetically targeted inhibitors affect the KEAP1-NFE2L2-centered modules. (**a**) Transcriptome perturbation of the “lightcyan (putative redox)” module in A549 cells. Fold changes in expression associated with drug treatment (dataset-1) are shown for 63 core “lightcyan” module genes in the heat map. The color key is shown in the margin. The genes downregulated (group 1) or upregulated (group 2) by BET inhibitors are shown and are framed in red and blue, respectively. (**b**) Epigenomic perturbation by (+)-JQ1 treatment. The ATAC-seq patterns of *ALDH3A1* (upper) and *GSR* (lower) were visualized using IGV (autoscale). Yellow arrows indicate the ATAC peaks with dose-dependent changes. (**c**) The combination indexes (CI) for dual stimulation with auranofin and (+)-JQ1 in the H2228, H2126 and II-18 cell lines. The CI-Fa (fraction affected) plot is shown. The method used for CI calculation is described in the Methods section and in Supplementary Fig. S11. The color legend is shown in the margin. (**d**) The CI-Fa plots of dual stimulation with (+)-JQ1 and vorinostat. The color legend is shown in **c**.

**Figure 6:**
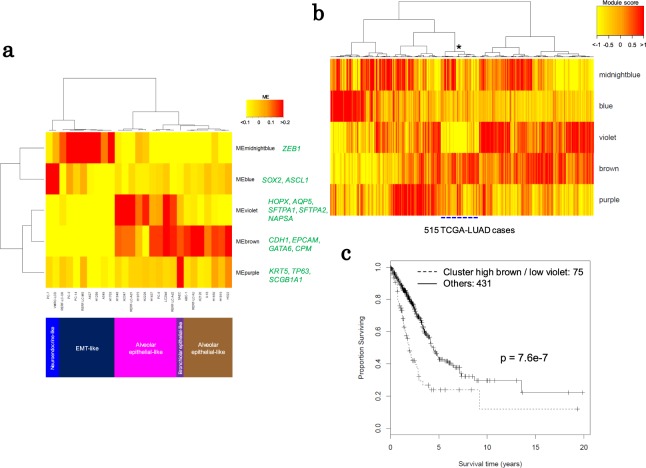
Module-based combinations for the stratification of lung cancer cell lines. (**a**) The lung cancer cell lines were clustered according to the module eigengenes of the five lineage-associated modules. Hierarchical clustering was performed using Ward’s method with Pearson correlation. The representative markers in the module are green. (**b**) TCGA-LUAD cases were classified according to the module scores of the five modules. The asterisk represents the cluster (75 cases are indicated by a dashed blue line) used in **c**. Hierarchical clustering of the module scores was performed using Ward’s method with Pearson correlation. (**c**) Kaplan-Meier analysis of the TCGA-LUAD cases divided into two groups according to the clustering of the module scores.

The strategic scheme used in this study is illustrated in Supplementary Fig. S1. Briefly, we induced perturbations in lung cancer cell lines using drugs and observed their consequences on multi-omics variables by using RNA-seq and ATAC-seq for transcriptome and epigenome analysis, respectively (Fig. 1). We simultaneously examined cell survival after drug treatment and evaluated the associations of drug sensitivities with the omics findings using conventional methods (Fig. 2). The collected multi-omics information was integrated into coregulated transcriptome modules (Figs. 3 and 4). We further characterized the modules and found that it was possible to control certain modules based on their landscape profiles (Fig. 5). Finally, we further utilized this approach to propose the module-based stratification of lung cancers for rational drug administration by targeting cell line-specific vulnerable modules (Fig. 6).

### High-throughput transcriptome and epigenome sequencing using drug-treated cell lines

To expedite data generation for the RNA-seq and ATAC-seq analyses, we developed and employed high-throughput RNA-seq and ATAC-seq procedures (Supplementary Fig. S2). First, for RNA-seq, we utilized the Fluidigm C1 single-cell library preparation system. After cell lysis, the extracted RNA was transferred to a separate reaction chamber in the C1 system, in which subsequencing reactions were performed. We found that more cost-effective library preparation was possible using the C1 microreaction chamber without degrading the quality of the sequencing templates. A similar procedure was developed and performed for ATAC-seq (see below).

We selected 95 compounds representing well-annotated approved drugs and various molecular-targeting drugs, including epigenetic drugs and approved receptor tyrosine kinase inhibitors. We collected detailed information for four concentrations of 23 compounds after 24 h, 48 h, and 72 h. Therefore, our dataset comprised five cell lines treated with four concentrations each of 23 compounds at three time points (dataset 1) and 23 cell lines treated with a single concentration of 95 compounds at one time point (dataset 2) (Tables 1 and 2). Using dataset 1, we deeply examined the transcriptomic and epigenomic changes caused by the representative drugs, including kinase inhibitors, cytotoxic anti-cancer drugs and epigenetic targeting drugs. For the larger-scale screening of transcriptomic and epigenomic perturbation, we also analyzed dataset 2, which contained a larger number of compounds and cell lines. A full list of the compounds and the detailed experimental conditions are summarized in Supplementary Table S1.

**Table 1:** General analyses of the high-throughput RNA-seq.

Dataset	Cell line	Total data points^*^ (% passed QC)	Average numbers per data point		
			Mapped reads	%mapped	%intron in mapped
Dataset-1 (4 conc. × 3 time points)	A549	249 (87%)	1,455,347	68%	8%
	H1299	253 (89%)	1,275,695	69%	7%
	H1648	251 (88%)	1,319,718	68%	7%
	H2347	245 (86%)	1,414,144	70%	9%
	II-18	231 (81%)	1,433,442	72%	10%
Dataset-2 (1 conc. × 1 time point)	23 cells	2011 (91%)	1,295,897	72%	9%

^*^≥0.5 million total reads; spike in control within 2 sd; <15% intron reads.

conc.: concentration, sd: standard deviation.

**Table 2:** General analyses of the high-throughput ATAC-seq.

		Total data points^*^ (% passed QC)	Average numbers per data point			
			Mapped reads	%mapped	%chrM in mapped	MACS peaks
Dataset-1 (4 conc. × 3 time points)	A549	251 (87%)	11,286,126	79%	17%	35,355
	H1299	269 (93%)	5,821,704	69%	65%	17,184
	H1648	264 (92%)	5,943,835	71%	58%	18,571
	H2347	276 (96%)	4,752,301	70%	64%	26,166
	II-18	256 (89%)	5,838,263	70%	64%	18641
Dataset-2 (1 conc. × 1 time point)	23 cells	2077 (94%)	8,158,509	71%	57%	17,734

^*^≥10,000 MACS peaks.

conc.: concentration.

### Evaluation of the RNA-seq and ATAC-seq datasets

We obtained 3,240 data points for the RNA-seq analysis. The RNA-seq data for each compound comprised an average of 1,356,520 and 1,794,226 sequencing reads in dataset 1 and dataset 2, respectively (Table 1). For quality control and initial evaluation purposes, the mapped reads were required to comprise at least 0.5 million reads containing 15% or fewer intron-mapped reads with an average reads per kilobase million (rpkm) value within ±2 standard deviations (sd) of that of the spike-in control for all wells. After the initial filtration, we further compared the expression abundances (rpkm values) of the protein-coding genes within the datasets to validate the reproducibility of the expression profiles using genes with an rpkm value ≥2 (Figs. 1a and S3). We observed a reasonably high positive correlation between the RNA-seq datasets (r = 0.90, concordance correlation coefficient (ccc) = 0.90 within each plate; r = 0.88, ccc = 0.88 between plates for A549 cells). The data was also reasonably correlated with standard RNA-seq data (Illumina TruSeq) that was previously obtained^8^ (r = 0.76 and r = 0.73) (Supplementary Fig. S3c). We also found that the gene expression changes were precisely represented in individual cases. An example of the changes induced by an HDAC inhibitor (CUDC 101) is shown in Fig. 1b. We observed that fold expression changes were moderately correlated between the RNA-seq datasets (r = 0.31, ccc = 0.31). The limited fraction of the genes showed significant changes even under the treatment with epigenetic targeting drugs (see also Supplementary Fig. S4).

For the ATAC-seq analysis, we selected ATAC-seq datasets with >10,000 peaks detected using MACS2^28^, yielding 3,393 ATAC-seq datasets with an average of 8,158,509 sequencing reads. We calculated the tag densities (ppm values) for the promoter and enhancer regions to determine the chromatin accessibility. Similar to the RNA-seq analysis, we evaluated the reproducibility of the ATAC-seq analysis. We found that the tag densities of the promoters were correlated between dataset-1 and 2 under the control condition (r = 0.96, ccc = 0.95 within each plate, r = 0.86, ccc = 0.85 between plates of A549 cells) (Figs. 1c and S3). We examined the changes in open chromatin in drug-treated cells (r = 0.36, ccc = 0.32 for promoters; r = 0.38, ccc = 0.35 for enhancers between the datasets under the CUDC 101 treatment) (Fig. 1d). For the RNA-seq and ATAC-seq data, we also conducted saturation analysis of the sequencing depths to investigate whether the sequencing depths were adequate for high-throughput monitoring of drug perturbation. We confirmed that >1 M reads were enough to analyze the expression patterns in the high-throughput RNA-seq. For the ATAC-seq, the open chromatin patterns and the numbers of the detected promoters and enhancers were saturated at >2 M reads (Supplementary Fig. S5).

When we compared the RNA-seq data and the ATAC-seq data for the promoter regions, we found that they were moderately correlated (Spearman r = 0.36) (Fig. 1e). The inconsistencies between the transcriptome and epigenome data may indicate that the promoter regions likely included open chromatin regions that lacked enhancer activity under any particular condition. Additionally, other regulatory factors may have affected transcription such as the open chromatin status of the enhancer regions.

For the validation studies, we compared the RNA-seq profiles from this study with the CCLE data (Fig. 1f)^10^. To confirm the mode of action, as measured by our procedures, we also evaluated whether the transcriptome signatures represented in our datasets were consistent with the L1000 CMAP signatures^19^. Using the CMAP dataset as a reference, we confirmed that the transcriptome signatures of vorinostat were highly concordant with those of HDAC inhibitors in the CMAP dataset (Fig. 1g). These results suggest that our RNA-seq data accurately represented the transcriptome mode of action for a particular drug. By conducting qPCR validation, we investigated whether the changes in transcriptome and epigenome status during the drug treatments were accurately calculated by the sequencing and post sequencing analyses. The results of the qPCR experiments using the RNA-seq and ATAC-seq libraries were generally highly consistent with the results of the RNA-seq and ATAC-seq analyses (Fig. 1h,i; also see Supplementary Fig. S6). Based on this validation, we considered that the substantial collected datasets could sufficiently represent the cellular changes reflected in the multi-omics profiles in response to the given chemical compounds.

### Multi-omics and the phenotypes of the lung adenocarcinoma cell lines

To measure the effects of the drugs on cell survival rates, we used 320 compounds, including those used for the multi-omics perturbation analysis. We measured the viability of each drug-treated cell line (Fig. 2a and Supplementary Table S2). This high-throughput screening assay provided a high average Z’ factor of 0.80 (range: 0.59–0.90). Consistent with previous studies^10,11^, we could evaluate the expected correlations between the genomic mutations/multi-omics statuses and the sensitivity of the cells to a series of drugs, including the EGFR inhibitors (Fig. 2b), multi tyrosine kinase inhibitors (i.e., crizotinib and vandetanib) and epigenetically targeted drugs (i.e., BET inhibitors) (Supplementary Fig. S7).

Based on the analysis of 26 representative cancer-related genes, including oncogenes, tumor suppressor genes, chromatin remodeling factors, and oncogenic fusion genes^8^, we divided the cell lines into two groups according to the cellular IC_50_ values and then compared the multi-omics statuses of the 26 genes in the two groups. This analysis identified 17 combinations associated with cell survival and genome and expression signatures (left panel, Fig. 2c). Genomic *KEAP1* mutations were significantly enriched in cells sensitive to the MEK inhibitor PD0325901 (middle and right panels, Fig. 2c). In cancer cells, oncogenic MAPK signaling is aberrantly upregulated and may upregulate *NFE2L2* transcription^29^. KEAP1 generally regulates the transcription factor NFE2L2 via a ubiquitin proteasome pathway^30^. When KEAP1 is mutated and NFE2L2 signaling is stabilized, cancer cells may be more addicted to MAPK oncogenic and KEAP1-NFE2L2 oxidation reduction (redox) signals and may become sensitive to MEK inhibitors. The identified KEAP1-centered module was subjected to further detailed analyses, as described in the following section (see below).

We used RNA-seq and ATAC-seq to identify the modes of action of the drugs (Fig. 1). For more detailed characterization of the drugs, we should consider the complex multi-omics status of each patient, which has not been sufficiently considered; this would strongly affect the transcriptional responses to drugs. Below, we describe transcriptome module-based target selection for the rational administration of drugs to lung cancer patients who harbor diverse genomic, epigenomic, and transcriptomic characteristics. We confirmed that potentially informative influences of drug treatments were unrecognized when assessed according to the cell survival rates.

### Gene co-expression network analysis to identify gene expression modules

To integrate the omics responses with the cellular responses, which remained latent occasionally, we first evaluated the multi-omics profiles according to the transcriptional regulatory modules. Initially, based on the RNA-seq data for all cell lines included in the datasets of untreated conditions, we employed the weighted gene co-expression network analysis (WGCNA) method^31^. We identified 73 co-expression modules within the transcriptional signatures (Fig. 3a, Supplementary Fig. S8 and Supplementary Table S3). We expected that individual genes with similar expression patterns, which were likely regulated by shared mechanisms, would be modulated. Thus, a bird’s eye-view analysis of the responses to drug perturbation would be possible. We selected 51 modules for further analyses after excluding 22 modules that were likely included mainly due to cell line-specific copy number aberrations. WGCNA analysis offers a major advantage because the value of the “module eigengene” (ME) may be used as an indicator to represent the activity of the corresponding module (Fig. 3b). The ME is calculated based on the first principal component of the expression patterns of the gene members included in the module.

### The antioxidative stress response module was associated with KEAP1-NFE2L2 mutations

We particularly focused on the “lightcyan” module, which may be controlled by a specific repertoire of drugs (Fig. 4a, see below). The “lightcyan” module comprised 158 genes, which were significantly associated with redox-related pathways enriched in “GO:0016616 oxidoreductase activity, acting on the CH-OH group of donors, NAD or NADP as acceptor” (Bonferroni p = 0.0013) (Supplementary Table S4). The “lightcyan” module included genes that respond to oxidative stressors such as *GSR* and *IDH1*^32^ (Supplementary Table S3).

To identify the transcription factor(s) controlling this module, we analyzed the ENCODE database^7^ to determine whether transcription factor bindings was enriched within the proximal regions of the 158 member genes. We found that *NFE2L2* may represent an associated transcription factor. The binding of NFE2L2 to the promoter or enhancer regions of 67 genes (44% of the “lightcyan” module members) was indicated by the ChIP-seq data from A549 cells in the ENCODE dataset (Fig. 4b). In lung adenocarcinoma cells, either the activation of NFE2L2 or the loss of its negative regulator KEAP1 frequently occur. We found that cell lines harboring *KEAP1* or *NFE2L2* mutations showed significantly increased module activity as represented by their module eigengene (p = 0.0001, Wilcoxon rank sum test) (Fig. 4c). For clinical specimens, we also retrieved and analyzed the RNA-seq and whole exome sequencing datasets from the TCGA^1^. We analyzed the 515 clinical samples of lung adenocarcinoma (TCGA-LUAD) and found that the activity of the “lightcyan” module was strongly correlated with the mutational status of *KEAP1* and *NFE2L2* (Fig. 4d).

We then analyzed the functional relevance of this module. NFE2L2 is a transcription factor that is activated by oxidative stress and controls the expression of genes that encode proteins that mediate downstream redox reactions, thereby providing robust activity against oxidative stress in cancer cells^33^. It was also essential to analyze metabolites for understanding these redox reactions. We measured abundances of 113 metabolites by metabolome profiling (see Methods). When we analyzed the metabolic profiles of the cell lines, we found that seven metabolites were significantly correlated with “lightcyan” module activity (Spearman r >0.65, p < 0.002). UDP-glucuronate (Spearman r = 0.84, Fig. 4e) serves as a substrate of UDP-glucose 6-dehydrogenase, which is encoded by *UGDH*, a member of the “lightcyan” module and a transcriptional target of NFE2L2. Further evidence that supports the conclusion that other metabolites are associated with this NFE2L2-centered module is presented in Supplementary Table S5. For modules which were associated with metabolic profiles, such as the “lightcyan” module, the genes encoding enzymes could be identified as the key genes of the modules. For these cases, we found that metabolites are, indeed, good indicators representing functional and biological relevance of the modules.

Finally, we conducted an NFE2L2 knockdown experiment in the A549 cell line. We directly confirmed the downregulation of several core members of the “lightcyan” module (Fig. 4f). The lines of evidence collectively indicated that the “lightcyan” module was an NFE2L2-centered module, and the activation of the “lightcyan” module was represented by the transcriptome signatures of *KEAP1*- and *NFE2L2*-mutant cancers.

To evaluate the clinical effects of controlling this module, we reanalyzed the TCGA datasets. Among a group of cancers (128 cases, 25%) with high levels of “lightcyan” module activity, significantly poor prognosis was observed (p = 0.019) (Fig. 4g). We further analyzed other cancer types and identified the same transcriptional dysregulation in cancers such as lung squamous cell carcinoma and esophageal carcinoma (198 and 35 cases; 39% and 19%, respectively) (Supplementary Fig. S9), indicating the general importance of this module in cancer. Therefore, effective drug intervention strategies may have broad clinical relevance.

### Drug interventions in the “lightcyan” module using auranofin

The activity of the “lightcyan” module was significantly correlated with sensitivity to auranofin treatment (r = 0.86, Spearman correlation coefficient) (Fig. 4a). Specifically, cell lines with increased expression levels of “lightcyan” module genes were resistant to auranofin. We sought to determine whether auranofin might be able to regulate the “lightcyan” module. Auranofin is used to treat rheumatoid arthritis. Auranofin is an inhibitor of thioredoxin reductase (encoded by *TXNRD1/2*)^32^. This drug is used as an approved drug to treat rheumatoid arthritis.

Figure 4h illustrates the association of antioxidative stress signaling with the “lightcyan” module. Auranofin may be associated with this module by regulating certain target genes of NFE2L2. However, in cell lines with increased “lightcyan” activity, other antioxidative stress genes and auranofin targets were also dysregulated, and these genes were not regulated by auranofin. Therefore, treatment solely with auranofin was insufficient for regulation of this module.

### BED inhibitors partially regulated the antioxidative stress response module

Then, we asked whether any additional compounds or combinations thereof could collectively regulate the dysregulated “lightcyan” module as a whole, since it could not be regulated by auranofin. Specifically, we determined whether any compounds altered the transcription of the remaining two submodules of the “lightcyan” module (Fig. 5a). Group 1 included genes downregulated by BET inhibitors, such as *GPX2* and *ALDH3A1*. On the other hand, genes upregulated by BET inhibitors were also detected in a different group, designated as group 2, which included *IDH1* and *GSR*.

We analyzed the transcriptome and epigenome perturbation datasets of the compounds with desirable profiles and found that the BET inhibitors (+)-JQ1 and GSK1210151A may be candidates that could downregulate the expression of group 1 genes (Fig. 5a). BET inhibitors belong to a class of so-called epigenome drugs that prevent the association between bromodomain proteins and acetylated histones and affect transcription. Our multi-omics catalog data showed that the expression levels of the *ALDH3A1*, *AKR1C3*, and *GPX2* genes were downregulated by BET inhibitors, which decreased the chromatin accessibility of their promoters or enhancers in a concentration-dependent manner (Figs. 5b and S10). We further investigated whether sensitivity to auranofin could be changed through transcriptional perturbations induced by BET inhibitors. We treated the cell lines with BET inhibitors combined with auranofin. In H2228 cells, which showed one of the highest activity levels in terms of the “lightcyan” module, auranofin and (+)-JQ1 showed synergistic activity (CI_50_ = 0.56) (Fig. 5c).

Despite the successful combined use of auranofin and (+)-JQ1, we found that the combination did not exert significant synergistic effects on some cell lines, such as H2126, II-18 and other cell lines (Figs. 5c and S11). We found that the combination of these compounds did not regulate the expression of group 2 genes (Fig. 5a). We additionally examined whether there were any other epigenetic drugs that could specifically control the expression of group 2 genes based on the observed omics information. As a candidate in place of auranofin, we used vorinostat with (+)-JQ1 treatment to more widely and moderately modulate the “lightcyan” module (Supplementary Fig. S12), and we observed a slight synergistic effect for (+)-JQ1 and vorinostat in some cells (CI_40_ = 0.61 in H2228 cells and CI_40_ = 0.80 in H2126 cells) with high activity in the “lightcyan” module (Fig. 5d).

To further elucidate the possible mode of action, we scrutinized the differences in the epigenome status between genes controlled and uncontrolled by BET inhibitors. For example, NFE2L2 binding sites reside in the enhancer region of *ALDH3A1*, which is one of the controlled genes. In this case, the chromatin structures represented in the ATAC-seq data were closed, and transcriptional activity was decreased by the BET inhibitors. In contrast, in *GSR*, the strong binding of NFE2L2 was localized to the promoter region, and the BET inhibitor did not close the associated chromatin structure, although a previously unknown enhancer located in the gene body (Fig. 5b) was inaccessible. These findings suggest that the regulatory mechanisms of NFE2L2 binding may be promoter- and enhancer-specific. In another example, *OSGIN1* was significantly upregulated in response to BET inhibitors (Figs. 5a and S10). *OSGIN1* is an oxidative stress response gene that is associated with the regulation of apoptosis. A regulatory region resides 25 kb upstream of this gene. We found that the chromatin structure was closed in response to treatment with BET inhibitors. When we analyzed the ENCODE ChIP-seq data, we found that this region overlapped with the binding site of the CTCF/cohesin complex^34^. The BET inhibitors may have changed the accessibility of the insulator regions, which may have affected the chromatin structure and transcriptional activity. We suggest that improving the understanding of the transcriptomic and epigenomic characteristics might help us to completely regulate the module.

Finally, we named the “lightcyan” module the “redox” module which was associated with *KEAP1*-*NFE2L2* dysregulation, metabolic features of antioxidative pathways and auraofin sensitivity and could be partly regulated by epigenetic inhibitors.

### Possible cell type-specific vulnerable modules

Overall, the above results indicate that the possible vulnerable modules, which should serve as the most effective targets for drug interventions, may differ depending on the cell type. Even when the putative redox module is not fully regulated in some cell lines, other modules may be targeted by other drug combinations. To further address this issue, we reorganized and characterized each of the lung cancer cell lines according to the patterns of the modules. We found that certain modules were associated with the lineages and differentiated phenotypes of the cells. We identified five modules that included representative markers of the cellular lineages and differentiated phenotypes. For example, the “brown” module included genes encoding epithelial markers such as E-cadherin (*CDH1*), *EPCAM*, *GATA6* and *CPM*^35^. The “violet” module included the alveolar type-I and -II cell markers *HOPX* and *AQP5* and the surfactant protein A genes (*SFTPA1*, *SFTPA2*, and *SFTA2*)^36^. The “violet” module also contained genes associated with the inflammatory response.

In contrast, the “midnightblue” module, which showed a significantly negative correlation with the “brown” module (r = −0.89, p = 4.0e-10, Pearson correlation coefficient) included the epithelial-mesenchymal transition regulator ZEB1^37^. The “purple” and “blue” modules included bronchiolar markers of basal and neuroendocrine cells^38,39^. Based on the activity patterns of these characteristic modules, we clustered the cell lines depending on their omics signatures (Fig. 6a).

Unlike classifications based on representative surface markers and genomic mutations, this module-based classification should reflect system-level cancerous aberrations. Indeed, when we mapped the activities of the modules to the clinical samples, the clinical samples of lung cancers exhibited diverse module characteristics (Fig. 6b). For example, the patterns of module activity were occasionally associated with the degree of malignancy, as indicated by the presence of poor prognosis (Fig. 6c). Moreover, *in vivo* cancers frequently showed remarkably diverse variations, although their driver mutations were the same. Distinct therapeutic strategies may be required depending on the unique multi-omic and phenotypic characteristics of different cancer types. To develop a new drug for each of these cancer subtypes, appropriate cell lines should be selected as models. It follows that the reclassification of cancers according to their gene expression modulations, in addition to the used of the current simple classification scheme based on their genomic mutational patterns, should provide clues regarding the mechanisms of oncogenesis and tumor progression that could be pharmacologically targeted.

## Methods

### Cell lines

Human lung cancer cell lines were cultured and harvested as previously described^8^. They are described in our database DBKERO (https://kero.hgc.jp/)^22^. For high-throughput RNA-seq and ATAC-seq, cells (1,500–12,000 cells per well) were seeded and treated with compounds in 96-well culture plates and then cultured for 24–72 h.

### Chemical compounds

Cells were treated with 320 compounds in the viability test. For this purpose, we selected 305 compounds from the Library of Pharmacologically Active Compounds (LOPAC, Sigma-Aldrich), 10 epigenetically targeted drugs and 4 tyrosine kinase inhibitors.

We designed high-throughput RNA-seq and ATAC-seq analyses that utilized drug treatment in a 96-well plate format. Two types of drug plates were prepared: dataset 1 included 24 compounds (23 compounds +a DMSO control) at four concentration points, and dataset 2 included 96 compounds (95 compounds +a DMSO control) at a single concentration and time point. For the RNA-seq and ATAC-seq analyses, 23 and 95 compounds, respectively, were selected from among the same compounds used for the viability test. According to their functional categories and growth inhibition rates, the compound showing some changes on cellular phenotypes were mainly selected. Some compounds were additionally selected from the FDA-approved Drug Library (Selleck Chemicals and Prestwick Chemical). The complete list is shown in Supplementary Table S1.

### High-throughput RNA-seq

The culture medium was discarded from a 96-well culture plate kept on ice. The cells were washed using cold PBS. Total RNA was extracted using TRIzol (Invitrogen). Briefly, 100 μl TRIzol was added to each well, and the cells were lysed. We prepared a new 1.5 ml tube with 400 μl TRIzol and transfered the lysed cells into the tube. For RNA separation, 100 μl chloroform was added and mixed well, and the sample was incubated at room temperature for 10 min. After the incubation, the sample was centrifuged (12000 rpm, 15 min, 4 °C), and the supernatant was transfered to a new 1.5 ml tube. Isopropanol precipitation was performed for RNA purification. The RNA sample was eluted in 10 μl nuclease-free water.

To perform the automatic reverse transcription and amplification reactions in microchambers, we used the C1 Single-Cell Auto Prep System (Fluidigm) with a modified script (provided at https://kero.hgc.jp/). The detailed procedure is shown in the Supplementary Methods. Briefly, reverse transcription and cDNA amplification were performed using the C1 Integrated Fluidic Circuit (C1 Single-Cell Auto Prep IFC for Open App, Fluidigm) with the C1 system. The purified cDNAs were used for library construction with the Nextera XT DNA Sample Preparation Kit (Illumina). The library was sequenced with a 35-base single-end run using the HiSeq. 2500 System (Illumina).

### High-throughput ATAC-seq

We developed a 96-well format ATAC-seq procedure according to the original ATAC-seq protocol^27^ with some modifications. The detailed procedure is shown in the Supplementary Methods. Briefly, the cells were dissociated using 0.25% Trypsin-EDTA (25200–056, Gibco) or Accutase^TM^ (A11105-01, Gibco) and pipetted into 8-channel tubes. For cell lysis, 50 μl lysis buffer (10 mM Tris-HCl pH 7.4, 10 mM NaCl, 3 mM MgCl_2_, and 0.1% NP-40) was used. For the ATAC reaction, 10 μl of 2 × TD buffer, 1 μl of Tn5 transposase and 4 μl of nuclease-free water were added, and the sample was incubated at 37 °C for 30 min. The transposed DNAs were purified using the ZR-96 DNA Clean & Concentrator-5 (Zymo Research) and eluted with 25 μl of nuclease-free water. PCR amplification was performed using 19.7 μl of transposed DNAs according to the original ATAC-seq protocol^27^, and then 10 μl of the sample in each of the 96 wells was mixed and purified. The libraries were sequenced with a 35-base single-end run using the HiSeq. 2500 platform (Illumina).

### ATAC-seq under basal conditions

To evaluate the open chromatin patterns of lung cancer cell lines under basal conditions, approximately 5 × 10^4^ cells were prepared and treated according to the ATAC-seq protocol previously described^27^.

### Determination of the promoter and enhancer regions

To determine the promoter regions, we selected the representative transcriptional start sites (TSSs) according to the start positions of the RefSeq transcripts of each gene using TSS-seq data (accession number DRA005903)^21^. We determined 13,431 promoter regions located within ±1.5 kb from representative TSSs.

To define the enhancer regions, H3K27ac and H3K4me1 ChIP-seq data from 27 cell lines were used (accession numbers DRA001860 and DRA002311)^8^. The MACS2^40^ peaks from the 27 cell lines were merged and the promoter regions were removed using bedtools^41^. We identified 312,226 and 535,538 regions according to the H3K27ac and H3K4me1 ChIP-seq peaks, respectively. All regions were merged using bedtools, and 515,599 regions were identified as enhancer regions. We used 56,961 enhancer regions that overlapped the ATAC-seq peaks under basal conditions. We assigned 32,111 enhancer regions to genes (±100 kb from TSSs).

### Analysis of the high-throughput RNA-seq and ATAC-seq data

RNA-seq tags were mapped to the reference genome (UCSC hg19) using ELAND software (Illumina). The tags were mapped to the sequences obtained from the three RNA spike in controls. The rpkm values were calculated for 22,657 genes and the spike-in controls. For the quality control of the RNA-seq data, three criteria were used: 1) >0.5 million mapped reads, 2) <15% intron mapped reads, and 3) rpkm values for the spike-in control that were within ±2 sd of the average for each plate. For the analysis of reproducibility, we used protein-coding genes with rpkm values ≥2 in both datasets. The ATAC-seq tags were mapped to the reference genome (UCSC hg19) using ELAND software (Illumina). The peaks were detected using MACS2 with the default parameters. ATAC-seq data with >10,000 MACS peaks were used for further analysis. The ppm values were calculated for the promoter and enhancer regions. As ELAND software is a somewhat older software, we compared the mapping results from ELAND with those from other tools and confirmed that there were no significant differences among the results of the mapping tools for the analysis of the high-throughput RNA-seq and ATAC-seq data (Supplementary Fig. S3d,g).

### CCLE RNA-seq data

For the lung adenocarcinoma cell lines A549 and H1299, RNA-seq data (CCLE_RNAseq_081117.rpkm.gct) were downloaded from the CCLE download page (https://portals.broadinstitute.org/ccle/data)^10^. The rpkm values were log2-tranformed after +1. For the comparison of the datasets obtained in this study, we used genes with rpkm values ≥2 in both datasets.

### qPCR validation of RNA-seq and ATAC-seq data

For the cDNAs that were used in the high-throughput RNA-seq analysis, qPCR validation was performed using THUNDERBIRD SYBR qPCR Mix (QPS-201, TOYOBO). We performed one assay for each measurement. The detailed procedure is shown in the Supplementary Methods. The primer sequences are listed in Supplementary Table S6.

We also performed qPCR analyses of the five promoters and eight enhancers for the validation of the high-throughput ATAC-seq data. We performed one qPCR assay for each measurement. The ATAC DNAs, which were subjected to sequence analysis, were used. The detailed procedure is shown in the Supplementary Methods. The primer sequences are listed in Supplementary Table S7.

### Cell viability test

Viability tests of cells treated with 320 drugs were performed using a 384-well plate high-throughput system. Each well of a 384-well plate contained 7.5 μl of culture medium. DMSO control wells were prepared with or without cells to serve as positive and negative controls, respectively. For high-throughput stimulation, 2.5 μl of a chemical compound was added to each well of the plates. For each well, 450–3,000 cells (15 μl of a cell suspension) were seeded and incubated for 72 h at 37 °C in an atmosphere containing 5% CO_2_. After incubation, 25 μl of the reagent provided with the CellTiter-Glo Luminescent Cell Viability Assay Kit (Promega) was added. A Multidrop Combi (Thermo Fisher Scientific) and a HORNET-NX (Wako) automatically dispensed the assay components. The cell viability rates were calculated according to the luminescence, which was measured using an EnVision Plate Reader (PerkinElmer). The assays were repeated twice (N = 2). The data were fitted to the logistic equation using XLfit (IDBS) as follows:

$$y=A+\frac{B-A}{1+{(\frac{C}{x})}^{D}}$$

The values represented the variables as follows: *y*: % growth inhibition; *x*: concentration of the compound; *A*: minimum value of *y*; *B*: maximum value of *y*; *C*: EC50 value; *D*: Hill slope^42^.

### Analysis of the association between the basal genomic status and drug sensitivity

The 26 cell lines were divided into two groups according to the IC_50_ values of each compound to produce the maximum degree of separation in terms of the IC_50_ values between the groups (Fig. 2c). The IC_50_ values were adjusted to 10 μM when the growth inhibition rate did not reach 50% at a 10 μM concentration. We focused on the mutational status of 26 cancer-related genes^8^ and used Fisher’s exact test to analyze the association between the mutational status and the cell groups defined by the IC_50_ value. An aberrant status was defined according to the following: for oncogenes, mutants were defined if the cell lines harbored genomic mutations and the expression was >5 rpkm; for tumor-suppressor genes, mutants were defined if they harbored mutations or showed low expression levels that were <5 rpkm. For other cancer-related genes, such as those encoding chromatin factors and fusion genes, mutants were defined if they showed genomic mutations. For *CDKN2A*, the DNA methylation status of the p16^INK4A^ alternative promoter was considered. Finally, we extracted the gene-compound pairs with a >5-fold IC_50_ differences between the two cell groups and a significant enrichment of the mutational status of the gene in either group (p < 0.05, Fisher’s exact test).

### CMAP analysis

Using the high-throughput RNA-seq data, genes with >4 or <0.25-fold changes in terms of their expression levels were extracted as parts of the signatures of the transcriptome mode of action. We used an RNA-seq dataset from PC-9 cells treated with vorinostat (0.95 μM for 24 h). Of the 4,798 genes expressed in either stimulated or DMSO control cells (rpkm value >10 for RNA-seq; ppm >5 ppm from the promoter region for ATAC-seq), 163 upregulated and 146 downregulated genes were extracted and designated a part of the signature of vorinostat. To verify the signature, we accessed CMAP datasets^19^ using CLUE (https://clue.io/). We used the CLUE L1000 Query to evaluate the similarities between the signature of our dataset to that of the CMAP datasets.

### Weighted gene co-expression network

For the extraction of the gene co-expression modules, we used RNA-seq data (accession numbers DRA001846 and DRA002311) from 27 cultured cell lines (26 lung cancer cell lines and small airway epithelial cells) incubated under basal conditions. We extracted 73 modules using the WGCNA method^31^. The detailed procedure is shown in the Supplementary Methods. The complete list of the 73 module members is included in Supplementary Table S3. GO enrichment analysis of each module was performed using the WGCNA GOenrichmentAnalysis function. The top 10 GO terms ordered according to the Bonferroni corrected p-values (Bonferroni p < 0.1) are listed in Supplementary Table S4.

### Analysis of the NFE2L2 binding patterns using ENCODE data

To evaluate the NFE2L2 binding patterns, NFE2L2 ChIP-seq data (ENCSR584GHV and NCSR949BZP) were obtained from the ENCODE project^7^. The fastq files (ENCFF002EAK and ENCFF002ECM) were mapped to UCSC hg19 using Bowtie2 (v2.0.6)^43^. The sorted bam files were created using SAMtools^44^. The peaks were detected using MACS2 (v2.1.0.20150420).

### Metabolome analysis

For the metabolome analyses, capillary electrophoresis time-of-flight mass spectrometry (CE-TOFMS) was performed using an Agilent CE-TOFMS system^45^. The raw data were analyzed using MasterHands (ver. 2.17.0.10)^46^. For further analysis, the average concentrations (fmol/cell, n = 2) were used for each cell line. A not detected (N.D.) value was omitted if the compound was detected using the other measurement. Concentrations were defined as zero if the metabolites were not detected by either measurement.

To evaluate the association with the transcriptome modules, Spearman correlation coefficients for the ME values of the modules and the concentrations of the metabolites were calculated. We detected 113 metabolites in >10 cell lines.

### siRNA knockdown experiments

A549 cells were seeded (1500 cells/well in 96-well plates) and incubated for 24 h at 37 °C. *NFE2L2* siRNAs (Silencer Select *NFE2L2* s9491 and s9493, Applied Biosystems) and negative control siRNAs (Silencer Select Negative Control #1 siRNA, Applied Biosystems) were used for the transfection of the A549 cells. Lipofectamine RNAiMAX Transfection Reagent (Thermo Fisher Scientific) and the siRNAs were diluted in serum-free MEM (M7278, Sigma-Aldrich). For the preparation of the RNA-lipid complexes, diluted Lipofectamine and siRNAs were mixed and incubated for 5 min, and 10 μl of siRNA-lipid reagents was added to the cells.

To confirm the knockdown efficiency and determine the changes in the expression levels of the downstream genes, qRT-PCR was conducted (n = 3). For qRT-PCR, cDNAs were synthesized using the SuperPrep Cell Lysis & RT Kit for qPCR (SCQ-101, TOYOBO), and qPCR was performed using the THUNDERBIRD SYBR qPCR Mix (QPS-201, TOYOBO). The PCR primers are listed in Supplementary Table S8.

### Analysis of TCGA data

For The data from TCGA for lung adenocarcinoma (LUAD), lung squamous cell carcinoma (LUSC) and esophageal carcinoma (ESCA), RNA-seq v2 data was acquired using TCGA-Assembler v2.0.6^47^ (the data checked on 2019/09/10). The clinical information was acquired from cBioPortal (downloaded on 2019/09/13). The somatic mutation data were downloaded from the NCI Genomic Data Commons (https://gdc.cancer.gov/) (downloaded on 2019/09/13-14). To generate the heat maps of the RNA-seq v2 data, the normalized counts were adjusted to 1 if the count was <1. The adjusted expression levels were normalized to the median expression levels in all patients and log2-transformed. Hierarchical clustering with Ward’s method was performed according to the Euclidean distance using the expression levels of the genes in the given modules.

To extract the core putative redox module member genes (Fig. 5a) that exhibited significant co-expression patterns in the clinical samples, we performed the Wilcoxon rank-sum test to compare the expression levels of TCGA-LUAD cases with high module activity (Fig. 4d) with those of other cases. We extracted 63 differentially co-expressed genes among the high module activity cases (Bonferroni p < 0.05).

To associate the TCGA transcriptome data with the modules, the “module score” for each TCGA-LUAD case was calculated for each module as follows: (1) the expression values (normalized counts) were log2-transformed after adding 1; (2) the log2-transformed values of 10 representative genes with the highest correlation with the ME were averaged for each case; (3) z-score normalization was applied. Kaplan-Meier analysis and the log-rank test were performed using the survival package in R.

### Treatment with a combination of auranofin and (+)-JQ1

For the combination treatment using auranofin and (+)-JQ1, the assay plate was designed as shown in Supplementary Fig. S11. The cell viability rates were calculated as described above. The combination index (CI) was calculated according to the Chou-Talalay method^48^.

### Treatment with a combination of vorinostat and (+)-JQ1

For the combination treatment using vorinostat and (+)-JQ1, the CI was calculated in a manner similar to that shown in Supplementary Fig. S11. By using three cell lines with high activity in the putative redox module, a cell viability test was performed twice, and the CI was determined according to the average cell viability rates.

## Supplementary information

Supplementary Methods and Figures

Supplementary Tables

## Data Availability

All the raw sequencing data used for the high-throughput RNA-seq and ATAC-seq analyses have been registered in DDBJ under the accession numbers DRA006875 - DRA006902 (RNA-seq) and DRA006903 - DRA006930 (ATAC-seq). The omics sequencing data for the cell lines were previously published^8,49^. The datasets in this paper are provided in our database, DBKERO (https://kero.hgc.jp/)^22^.
