# Supplementary material for: Characterization of cancer omics and drug perturbations in panels of lung cancer cells
Source: Sci Rep. 2019 Dec 20;9:19529. doi: 10.1038/s41598-019-55692-9 (PMC6925249; doi:10.1038/s41598-019-55692-9)
Supplement: Supplementary file 1 — Supplementary Methods and Figures [file 41598_2019_55692_MOESM1_ESM.pdf]

Characterization of cancer omics and drug perturbations in panels of lung cancer cells

Ayako Suzuki†, Keiichi Onodera†, Ken Matsui, Masahide Seki, Hiroyasu Esumi, Tomoyoshi Soga, Sumio Sugano, Takashi Kohno, Yutaka Suzuki and Katsuya Tsuchihara

Supplementary Methods (p. 2 - 5)

Supplementary Figures S1 - S12 (p. 6 - pp. 23)

References (pp. 24)

Supplementary Tables are provided in a separate Excel file.

†These authors contributed equally to this work.

Supplementary Methods

High-throughput RNA-seq

To perform the 96-well format high-throughput RNA-seq assay, we used the C1 Single-Cell Auto Prep System (Fluidigm) with a modified script (provided at https://kero.hgc.jp/) for the automatic reverse transcription and amplification reactions in microchambers. For the RNA spike-in control, we prepared RNA Spikes Mix (RNA Spikes #1, #4 and #7, ArrayControl RNA Spikes, Thermo Fisher Scientific) according to the user manual of the SMART-Seq v4 Ultra Low Input RNA Kit for the Fluidigm C1 System (Takara/Clontech). Three reagent mixtures were prepared using the SMART-Seq v4 Ultra Low Input RNA Kit for Sequencing (Takara/Clontech) and the C1 Single-Cell AutoPrep Reagent Kit for mRNA seq (Fluidigm) as follows: Mix A, 2.4 µl of RNA Spikes Mix (1/10), 168 µl of 3' SMART-Seq CDS Primer II A, 261.6 µl of nuclease-free water and 48 µl of 10× lysis buffer with RNase inhibitor; Mix B, 0.6 µl of C1 Loading Buffer, 8.2 µl of 5× Ultra Low First-Strand Buffer, 2.05 µl of SMART-Seq v4 Oligonucleotides, 1.05 µl of RNase inhibitor, and 4.1 µl of SMARTScribe Reverse Transcriptase; Mix C, 50 µl of 2× SeqAmp PCR Buffer, 2 µl of PCR Primer IIA, 2 µl of SeqAmp DNA Polymerase, 3 µl of nuclease-free water and 3 µl of C1 Loading Buffer. To prepare the C1 Integrated Fluidic Circuit (IFC) (C1 Single-Cell Auto Prep IFC for Open App, Fluidigm), Harvest Reagent and Blocking Reagent were added to the IFC. After priming, 7 µl of Cell Wash Buffer and 20 µl of Preloading Buffer were added. After the preparation of the C1 IFC, the sample (2 µl of total RNA sample and 4 µl Mix A) was prepared, and 5 µl of the sample was transferred into the C1 IFC. After automatic reverse transcription and amplification by the C1 system, the amplified cDNAs were collected from the C1 IFC and purified by Agencourt AMPure XP (Beckman Coulter). The purified cDNAs were used for library construction. The RNA-seq library was constructed using the Nextera XT DNA Sample Preparation Kit (Illumina) and

sequenced using a HiSeq 2500 System (Illumina).

High-throughput ATAC-seq

The culture medium was discarded from a 96-well plate (75 µl of CellBanker 1 plus was added to freeze the plates). The cells were washed with 100 µl of cold PBS. To dissociate the cells, 50 µl of 0.25% Trypsin-EDTA (25200-056, Gibco) or Acutase (A11105-01, Gibco) was added, and the plate was incubated at 37°C for 5 - 10 min. After dissociation, 150 µl of culture medium was added, and the cells were gently pipetted into 12 8-channel tubes. The cells were centrifuged at 300 ×g at 4°C for 5 min, and the supernatant was discarded without disturbing the precipitate. The cells were dissolved in 200 µl of PBS containing Complete Protease Inhibitor Cocktail (Roche) with gentle pipetting. The cells were centrifuged at 300 ×g at 4°C for 5 min, and the supernatant was discarded without disturbing the pelleted cells. For cell lysis, 50 µl lysis buffer (10 mM Tris-HCl pH 7.4, 10 mM NaCl, 3 mM $MgCl_2$, and 0.1% NP-40) was added, and the sample was mixed using a pipet. The cells were centrifuged at 300 ×g at 4°C for 10 min, and the supernatant was removed. For the ATAC reaction, 10 µl of 2× TD buffer, 1 µl of Tn5 transposase and 4 µl of nuclease-free water were added, and the sample was incubated at 37°C for 30 min. After the reaction was complete, the transposed DNAs were purified using the ZR-96 DNA Clean & Concentrator-5 (Zymo Research) and eluted in 25 µl of nuclease-free water in a new 96-well plate.

For PCR amplification, 19.7 µl of sample containing transposed DNA, 2.5 µl of customized Nextera PCR primer 1, 2.5 µl of customized Nextera PCR primer 2, 0.3 µl of 100× SYBR Green I, and 25 µl of NEBNext High-Fidelity 2× PCR Master Mix were mixed in a new 96-well PCR plate. The PCR was conducted as follows: 1 cycle of 5 min at 72°C and 30 s at 98°C; 5 cycles of 10 s at 98°C, 30 s at 63°C and 1 min at 72°C. Using 5 µl of the amplified samples contained in eight wells, real-time

PCR was performed to determine the additional number of cycles required for the PCR in a similar manner as previously described[1]. PCR amplification was performed using the remaining 45 µl of the amplified samples, and then 10 µl of sample in each of the 96 wells was mixed into a single tube and purified using a MinElute PCR Purification Kit (Qiagen). The ATAC DNA library was eluted in 20 µl of nuclease-free water. The libraries were sequenced using the HiSeq 2500 platform (Illumina).

qPCR validation of the RNA-seq and ATAC-seq data

For the validation of high-throughput RNA-seq data, qPCR was performed for representative genes (n = 1 for each assay) (**Fig. 1h**). By using the cDNAs that were used in the sequencing analysis, qPCR was performed using THUNDERBIRD SYBR qPCR Mix (QPS-201, TOYOBO). We selected 28 and 30 genes, respectively, (including *ACTB* as a housekeeping gene and *GAPDH* as a control) for each of the two treatments (0.89 µM (+)-JQ1 for 24 h and 0.82 µM CUDC 101 for 24 h). The genes were selected according to the following criteria: 1) genes with expression levels in the DMSO control >0; 2) the top 28 genes in terms of median fold changes in expression in treated A549, II-18, H2347 and H1648 cells. *FAM195B* and *RNF24* were not evaluated in (+)-JQ1-reated cells because we were unable to design primer sequences. Genes with a Ct value ≥35 or multiple peaks in the melting curve were not used. The primer sequences are listed in **Supplementary Table S6**.

To validate the high-throughput ATAC-seq data, we performed qPCR analyses of the five promoters and eight enhancers (n = 1 for each assay) (**Fig. 1i** and **Supplementary Fig. S6**). ATAC DNAs, which were subjected to sequence analysis, were used. To validate the differences in chromatin accessibility among the cell lines, qPCR experiments were conducted for the *EGFR* promoter and enhancer and the *MYC* enhancer in 22 cell lines (except for H1648 cells, which failed quality control testing by using the ATAC-seq data) using DMSO control samples (upper panel, **Fig.**

1i). To confirm the chromatin changes resulting from (+)-JQ1 treatment (0, 0.01, 0.1, 1, 10 μM; 24 h), qPCR was used to analyze the regulatory regions of *ALDH3A1*, *GSR*, *GPX2* and *OSGIN1* (lower panel, **Fig. 1i**) in A549 cells. For CUDC 101 treatment (0, 0.01, 0.1, 1 μM; 24 h), we conducted qPCR analyses of the *ETV1* (lower panel, **Supplementary Fig. S6**) and *GYG1* enhancer regions in A549 cells. *GAPDH* served as a control[2] for the analysis of the fold changes in intensities (upper panel, **Supplementary Fig. S6**). The primer sequences are listed in **Supplementary Table S7**.

Weighted gene co-expression network

We used RNA-seq data (accession number DRA001846) from 27 cultured cells. The expression levels (rpkm values) of 21,730 protein-coding genes were calculated as previously reported[3]. Using TSS-seq data, 13,407 genes with ppm >5 in ≥1 cell line(s) were selected for the analysis. The rpkm values were log2-transformed with +1 adjustment and used as the input. For the extraction of the gene co-expression modules, the WGCNA method was used, and the soft thresholding power was selected (**Supplementary Fig. S8**)[4]. Using the WGCNA blockwiseModules function, 73 modules comprising 12,346 assigned genes (range: 30 - 2835) were extracted using the following parameters: networkType="signed", power=11, deepSplit=2, maxBlockSize=15000, minModuleSize=15, reassignThreshold =0, mergeCutHeight=0.25, detectCutHeight =0.995, TOMType ="signed". We defined 1,061 genes as the outliers of the modules. The module eigengenes calculated based on the first principal component of the module were used to determine the activities of the modules in each cell line. We excluded 22 modules from the analysis because >40% of the genes in each were located on the same chromosome, which could be caused by cell-specific copy number aberrations.

Supplementary Figures

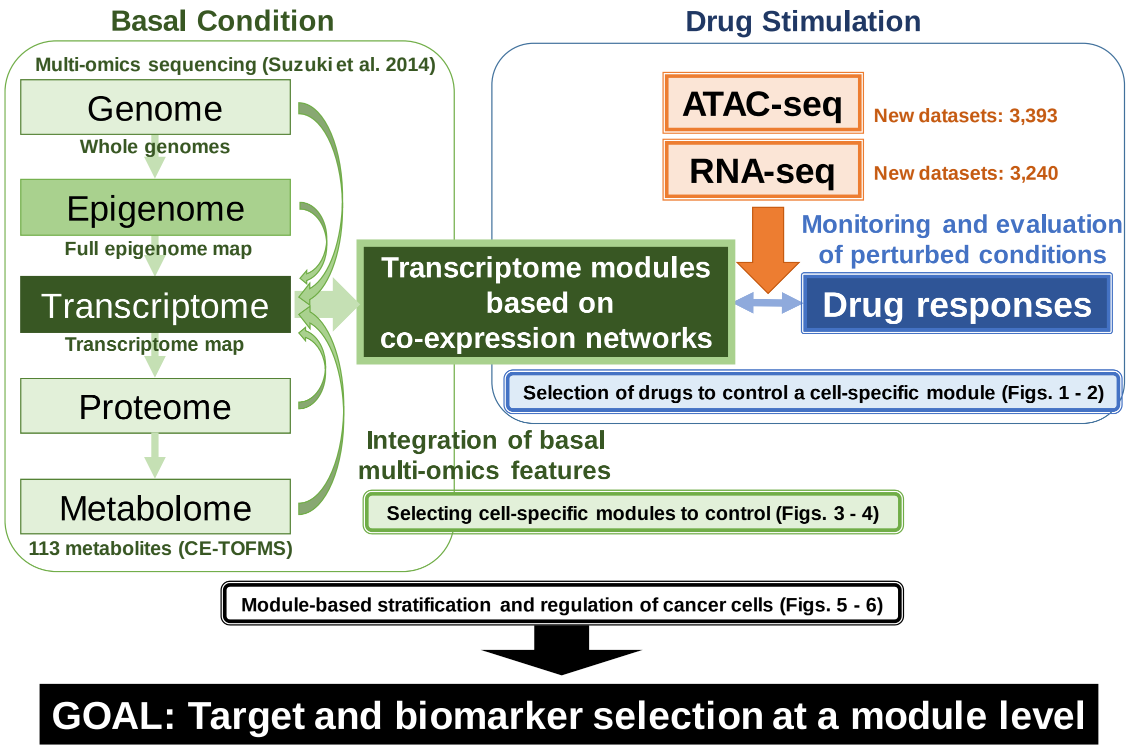

Supplementary Figure S1. Module-based stratification and regulation of lung cancer cell lines.

High-throughput RNA-seq and ATAC-seq analyses of drug-treated cells were performed, and the transcriptome modules were identified from a multi-omics sequencing catalogue to detect the targets of drug treatment at a module level. Ultimately, rational combinations of drugs and targets/biomarkers (modules) could be selected.

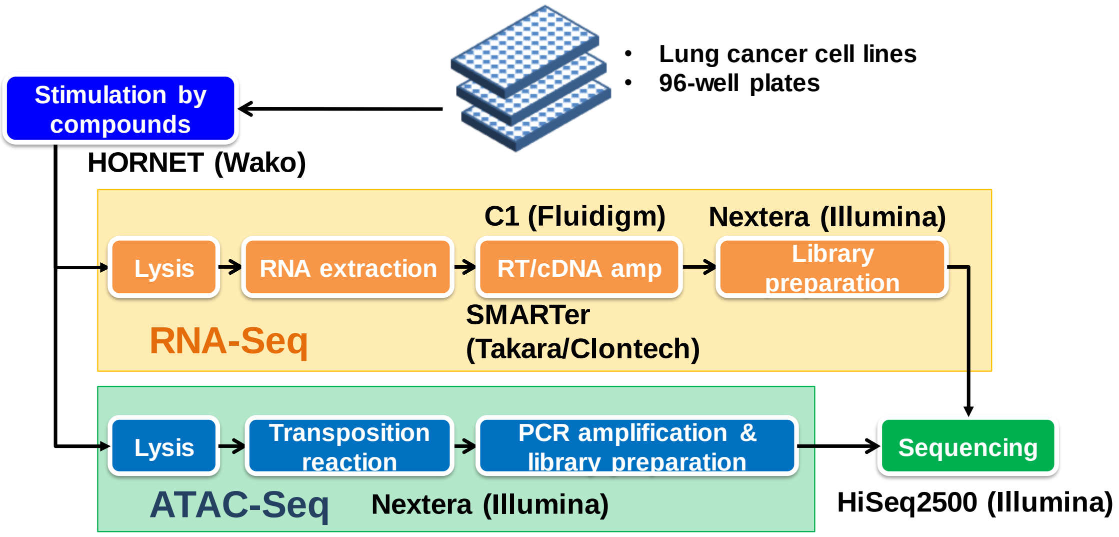

Supplementary Figure S2. The procedure used for high-throughput RNA-seq and ATAC-seq.

The workflow for high-throughput RNA-seq and ATAC-seq developed to monitor the transcriptome and epigenome perturbations in drug-treated cells. Cancer cell lines were seeded into 96-well culture plates and treated with the compounds. For RNA-seq, total RNA was extracted. Automatic reverse transcription and amplification were performed in microchambers with the C1 system. For ATAC-seq, we constructed a 96-well-based library via modification of the original ATAC-seq protocol[1]. Sequencing was performed using the HiSeq platform.

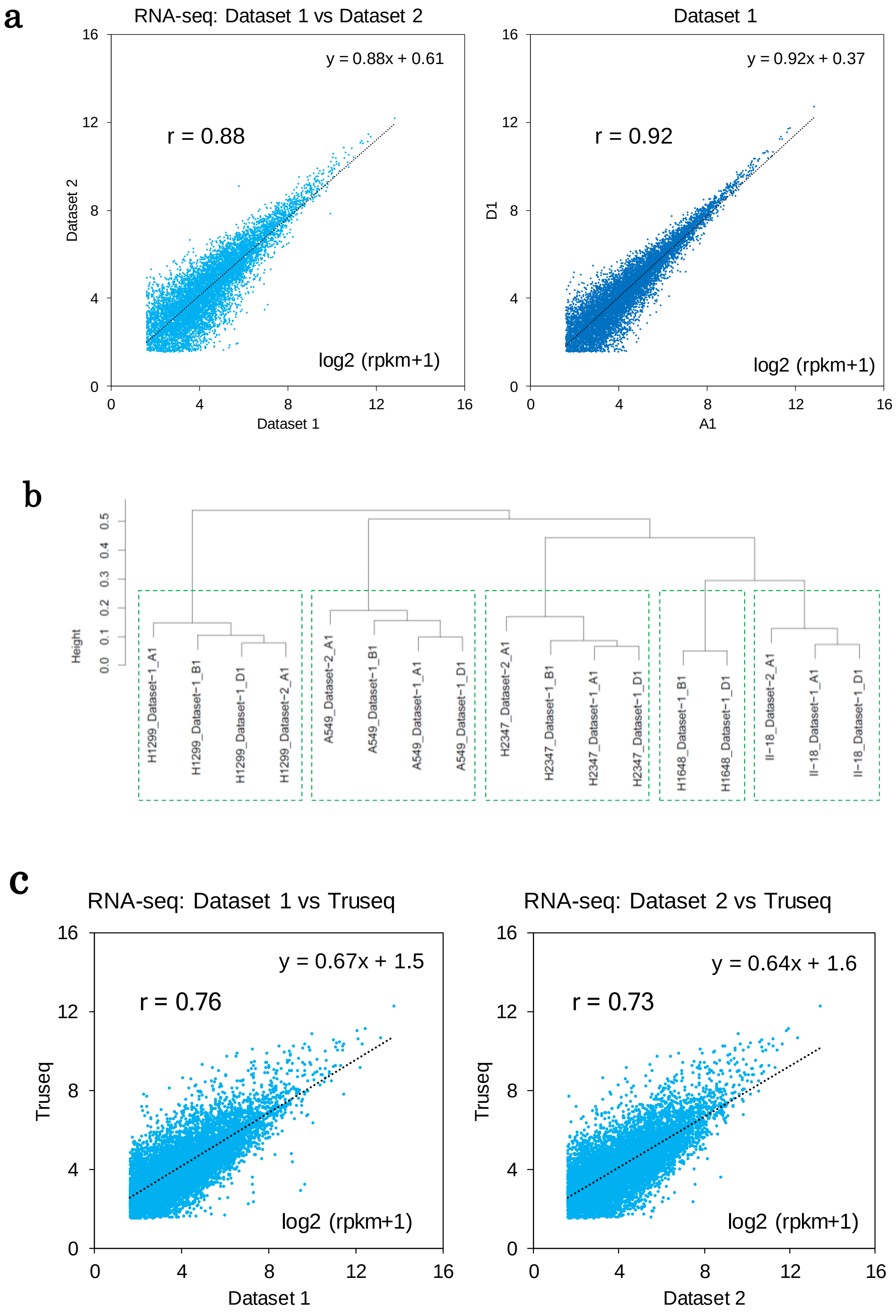
a
RNA-seq: Dataset 1 vs Dataset 2
y = 0.88x + 0.61
r = 0.88
log2 (rpkm+1)
Dataset 1
Dataset 2
Dataset 1
y = 0.92x + 0.37
r = 0.92
log2 (rpkm+1)
A1
D1
b
Height
H1299_Dataset−1_A1
H1299_Dataset−1_B1
H1299_Dataset−1_D1
H1299_Dataset−2_A1
A549_Dataset−2_A1
A549_Dataset−1_B1
A549_Dataset−1_A1
A549_Dataset−1_D1
H2347_Dataset−2_A1
H2347_Dataset−1_B1
H2347_Dataset−1_A1
H2347_Dataset−1_D1
H1648_Dataset−1_B1
H1648_Dataset−1_D1
Il−18_Dataset−2_A1
Il−18_Dataset−1_A1
Il−18_Dataset−1_D1
c
RNA-seq: Dataset 1 vs Truseq
y = 0.67x + 1.5
r = 0.76
log2 (rpkm+1)
Dataset 1
Truseq
RNA-seq: Dataset 2 vs Truseq
y = 0.64x + 1.6
r = 0.73
log2 (rpkm+1)
Dataset 2
Truseq

d

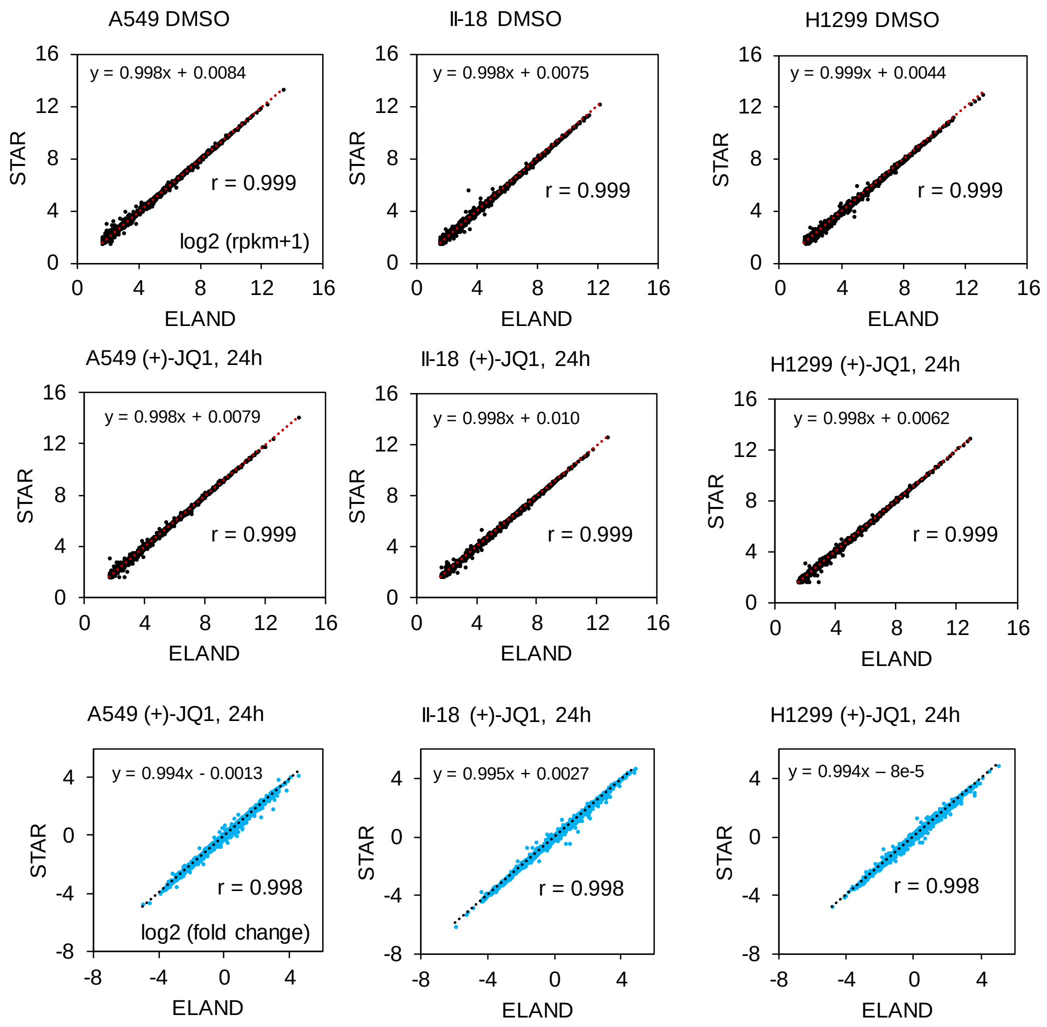

A549 DMSO
y = 0.998x + 0.0084
r = 0.999
log2 (rpkm+1)
STAR
ELAND
Il-18 DMSO
y = 0.998x + 0.0075
r = 0.999
H1299 DMSO
y = 0.999x + 0.0044
r = 0.999
A549 (+)-JQ1, 24h
y = 0.998x + 0.0079
r = 0.999
Il-18 (+)-JQ1, 24h
y = 0.998x + 0.010
r = 0.999
H1299 (+)-JQ1, 24h
y = 0.998x + 0.0062
r = 0.999
A549 (+)-JQ1, 24h
y = 0.994x - 0.0013
r = 0.998
log2 (fold change)
Il-18 (+)-JQ1, 24h
y = 0.995x + 0.0027
r = 0.998
H1299 (+)-JQ1, 24h
y = 0.994x – 8e-5
r = 0.998

e

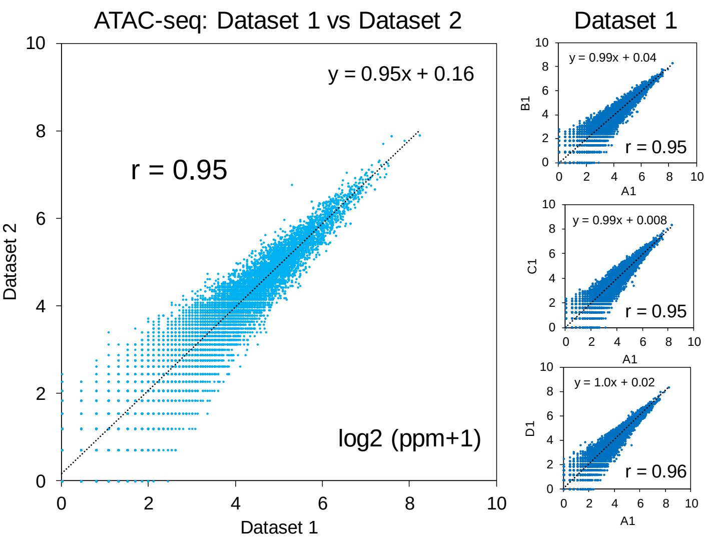

ATAC-seq: Dataset 1 vs Dataset 2
y = 0.95x + 0.16
r = 0.95
log2 (ppm+1)
Dataset 2
Dataset 1
Dataset 1
y = 0.99x + 0.04
r = 0.95
B1
A1
y = 0.99x + 0.008
r = 0.95
C1
A1
y = 1.0x + 0.02
r = 0.96
D1
A1

f

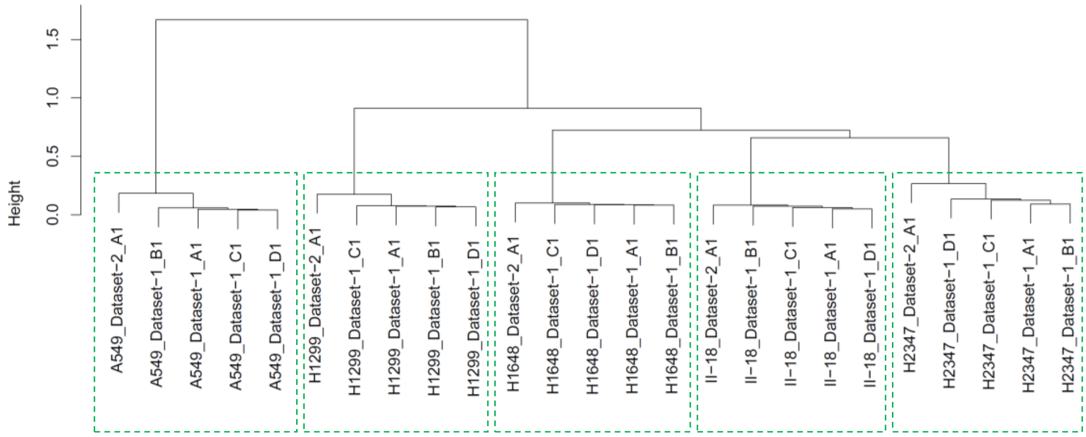
Height
0.0
0.5
1.0
1.5
A549_Dataset−2_A1
A549_Dataset−1_B1
A549_Dataset−1_A1
A549_Dataset−1_C1
A549_Dataset−1_D1
H1299_Dataset−2_A1
H1299_Dataset−1_C1
H1299_Dataset−1_A1
H1299_Dataset−1_B1
H1299_Dataset−1_D1
H1648_Dataset−2_A1
H1648_Dataset−1_C1
H1648_Dataset−1_D1
H1648_Dataset−1_A1
H1648_Dataset−1_B1
Il−18_Dataset−2_A1
Il−18_Dataset−1_B1
Il−18_Dataset−1_C1
Il−18_Dataset−1_A1
Il−18_Dataset−1_D1
H2347_Dataset−2_A1
H2347_Dataset−1_D1
H2347_Dataset−1_C1
H2347_Dataset−1_A1
H2347_Dataset−1_B1

g

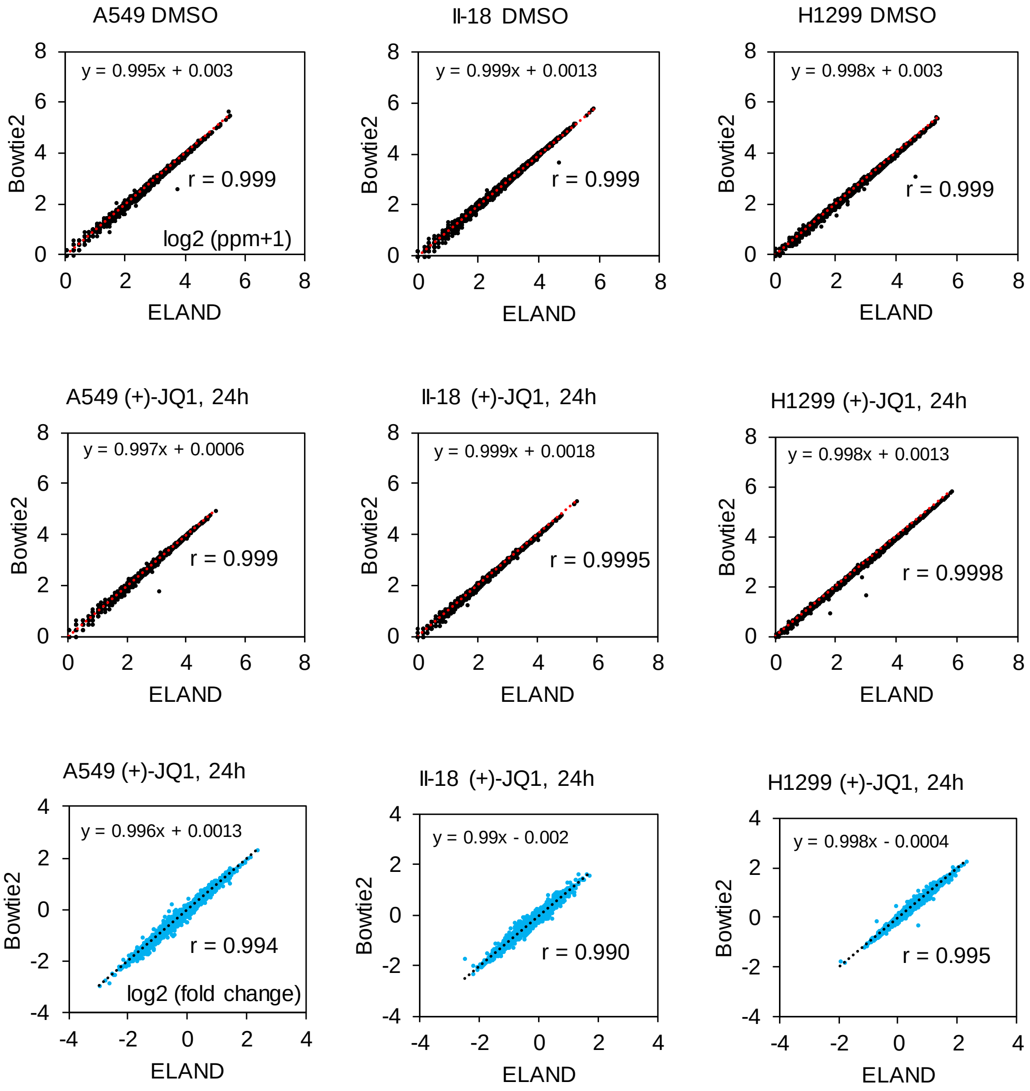
A549 DMSO
y = 0.995x + 0.003
r = 0.999
log2 (ppm+1)
Il-18 DMSO
y = 0.999x + 0.0013
r = 0.999
H1299 DMSO
y = 0.998x + 0.003
r = 0.999
A549 (+)-JQ1, 24h
y = 0.997x + 0.0006
r = 0.999
Il-18 (+)-JQ1, 24h
y = 0.999x + 0.0018
r = 0.9995
H1299 (+)-JQ1, 24h
y = 0.998x + 0.0013
r = 0.9998
A549 (+)-JQ1, 24h
y = 0.996x + 0.0013
r = 0.994
log2 (fold change)
Il-18 (+)-JQ1, 24h
y = 0.99x - 0.002
r = 0.990
H1299 (+)-JQ1, 24h
y = 0.998x - 0.0004
r = 0.995
Bowtie2
ELAND

Supplementary Figure S3. Reproducibility of the datasets obtained from the high-throughput sequencing platform.

(**a**) The rpkm values were compared in the datasets within or between plates (dataset 1 and 2) of II-18 cells in a similar manner as that shown in **Figure 1a** (r = 0.92, ccc = 0.92 within each plate; r = 0.88, ccc = 0.87 between plates). B1 in dataset 1 is not shown because of QC failure. (**b**) Hierarchical clustering analysis of the 17 RNA-seq datasets (DMSO control) among the five cell lines (Pearson correlation, Ward's method). The rpkm values of the protein-coding genes (rpkm >5 in ≥1 cell(s)) were used. The datasets from the same cell lines were classified in the same clusters. (**c**) The rpkm values were compared between dataset 1 (left) or dataset 2 (right) and published Truseq RNA-seq data. The rpkm values were adjusted by +1 and log2-transformed. The Pearson correlation coefficient is shown in the inset. (**d**) Comparison of the RNA-seq results generated by different mapping tools (ELAND and STAR[5]). We used sixdatasets (DMSO and (+)-JQ1 in A549, II-18 and H1299) for the comparison. The results of the comparison of rpkm values and fold changes were shown in top/middle and bottom panels, respectively. (**e**) The ppm values were compared in ATAC-seq datasets within or between plates (dataset 1 and 2) of II-18 cells in a similar manner as that shown in **Figure 1c** (r = 0.95, ccc = 0.95 within each plate; r = 0.95, ccc = 0.95 between plates). (**f**) Hierarchical clustering analysis of the 25 ATAC-seq datasets (DMSO control) among the five cell lines (Pearson correlation, Ward's method) in a similar manner as that shown in **b**. The ppm values of the promoter regions (ppm >5 in ≥1 cell(s)) were used. The datasets from the same cell lines were classified in the same clusters. (**g**) Comparison of the ATAC-seq results generated by the different mapping tools (ELAND and Bowtie2[6]) in a similar manner as that shown in **d**.

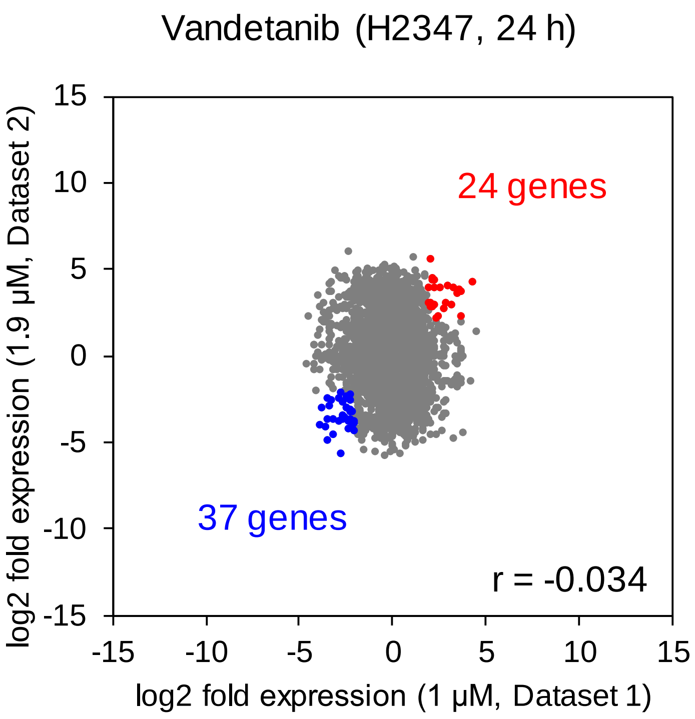

Supplementary Figure S4. Gene expression changes in cells treated with vandetanib. The same analysis shown in **Figure 1b** was conducted. Expression changes were calculated and compared between dataset 1 and dataset 2 from cells treated with vandetanib under similar conditions. The fold changes had no significant correlation between the two datasets (r = -0.034, ccc = -0.029) because there were less significant expression changes induced by vandetanib stimulation.

Note that the multi tyrosine kinase inhibitor vandetanib changed the expression levels of fewer genes than epigenetic inhibitors, while CUDC 101 (**Fig. 1b**) inhibited epigenomic factors such as histone deacetylases (HDAC) and more strongly affected the genome-wide epigenome and transcriptome patterns.

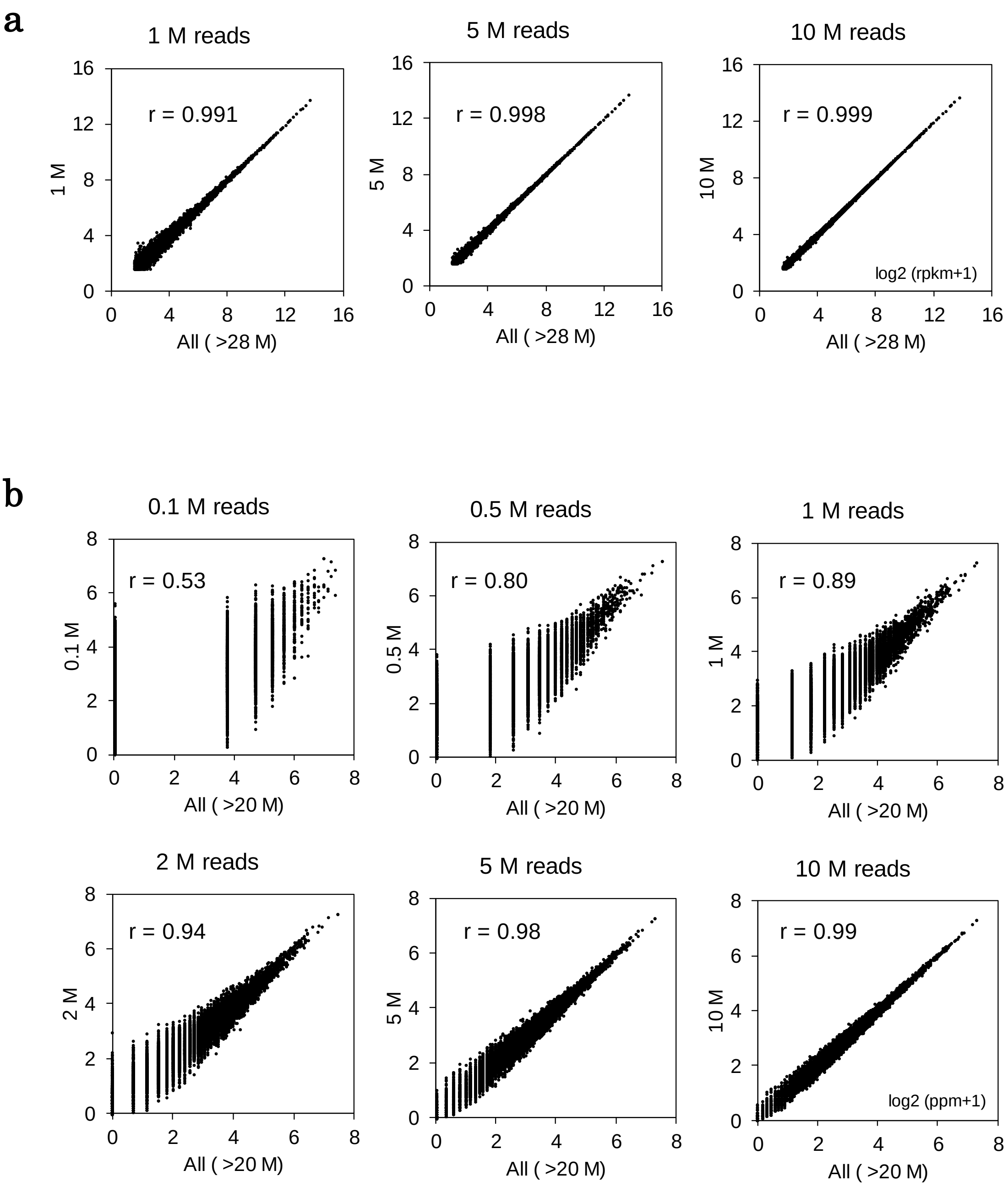
a
1 M reads
r = 0.991
1 M
All (>28 M)
5 M reads
r = 0.998
5 M
All (>28 M)
10 M reads
r = 0.999
10 M
log2 (rpkm+1)
All (>28 M)
b
0.1 M reads
r = 0.53
0.1 M
All (>20 M)
0.5 M reads
r = 0.80
0.5 M
All (>20 M)
1 M reads
r = 0.89
1 M
All (>20 M)
2 M reads
r = 0.94
2 M
All (>20 M)
5 M reads
r = 0.98
5 M
All (>20 M)
10 M reads
r = 0.99
10 M
log2 (ppm+1)
All (>20 M)

c

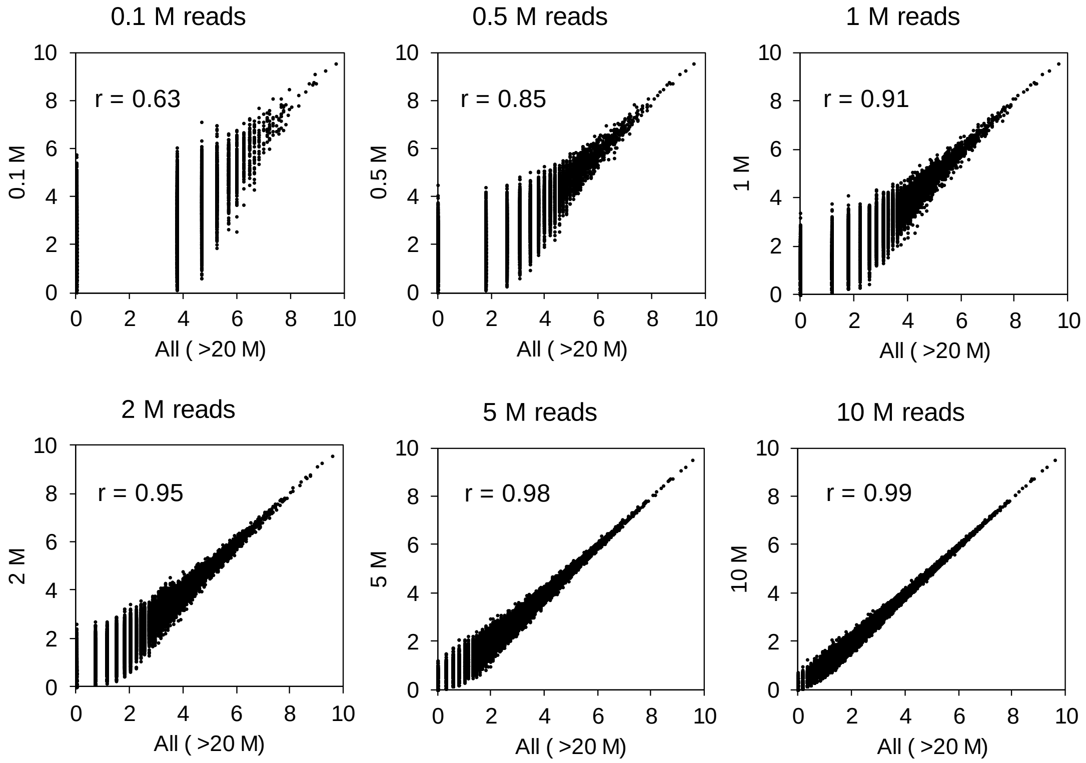
0.1 M reads
r = 0.63
0.1 M
All (>20 M)
0.5 M reads
r = 0.85
0.5 M
All (>20 M)
1 M reads
r = 0.91
1 M
All (>20 M)
2 M reads
r = 0.95
2 M
All (>20 M)
5 M reads
r = 0.98
5 M
All (>20 M)
10 M reads
r = 0.99
10 M
All (>20 M)

d

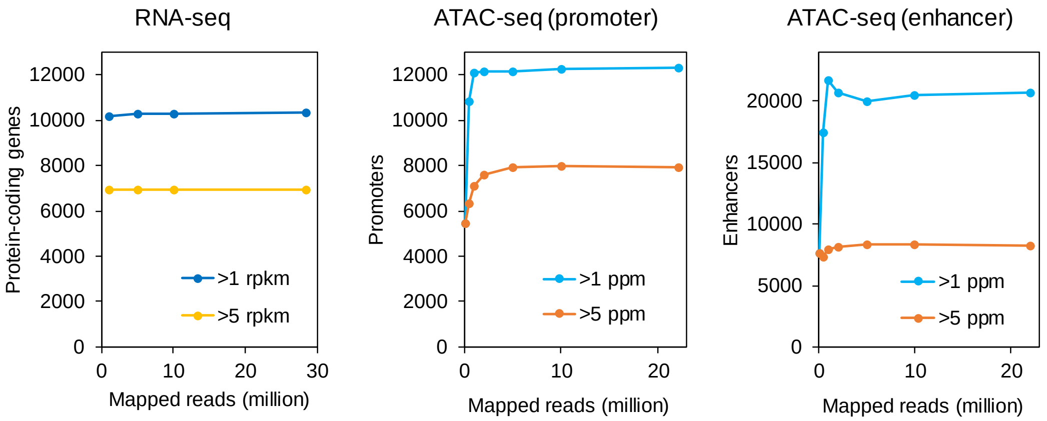
RNA-seq
Protein-coding genes
>1 rpkm
>5 rpkm
Mapped reads (million)
ATAC-seq (promoter)
Promoters
>1 ppm
>5 ppm
Mapped reads (million)
ATAC-seq (enhancer)
Enhancers
>1 ppm
>5 ppm
Mapped reads (million)

e

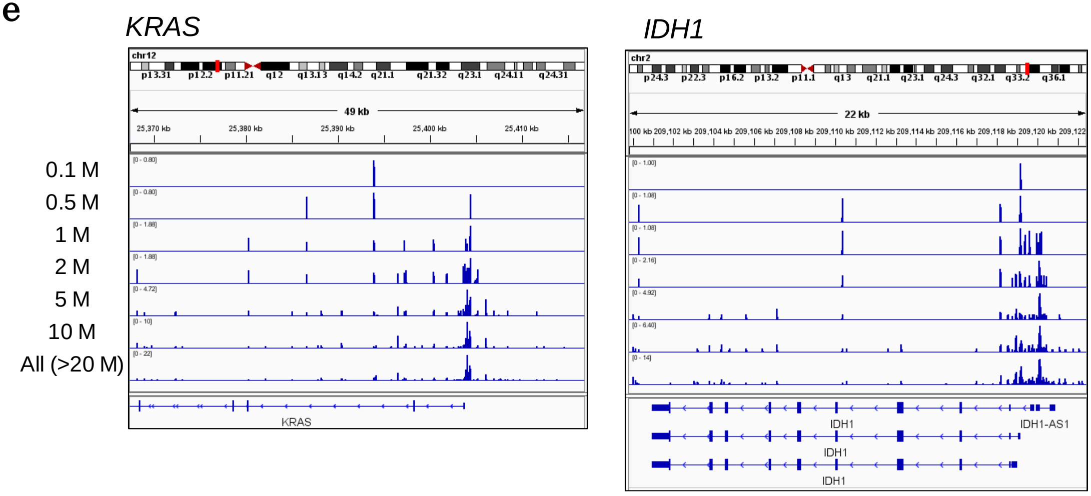

Supplementary Figure S5. Saturation analysis of the sequencing depths.

(**a**) Comparison of the expression patterns among the datasets with different sequencing depths of RNA-seq (A549, DMSO). We randomly selected reads for downsampling to 1 million, 5 million and 10 million reads. For the analysis, genes with rpkm ≥2 were used. The Pearson correlation coefficient is shown in the inset. (**b**) Comparison of the open chromatin patterns in the promoters of genes among datasets with different sequencing depths of ATAC-seq (A549, DMSO control). We randomly selected reads for downsampling to 0.1 million, 0.5 million, 1 million, 2 million, 5 million and 10 million reads. The Pearson correlation coefficient is shown in the inset. (**c**) Comparison of the open chromatin patterns in the 32,111 enhancers associated with genes among datasets with different sequencing depths of ATAC-seq in a similar manner as that shown in **b**. (**d**) The numbers of detected protein-coding genes (RNA-seq), promoters and enhancers (ATAC-seq) with different sequencing depths were shown in the left, middle and right panels, respectively. (**e**) Comparison of the peak patterns of ATAC-seq among the datasets with different sequencing depths (same as **b**). IGV visualizations were shown for *KRAS* (a driver gene for A549 cells) and *IDH1* (one of the member genes in the redox module) in left and right panels, respectively.

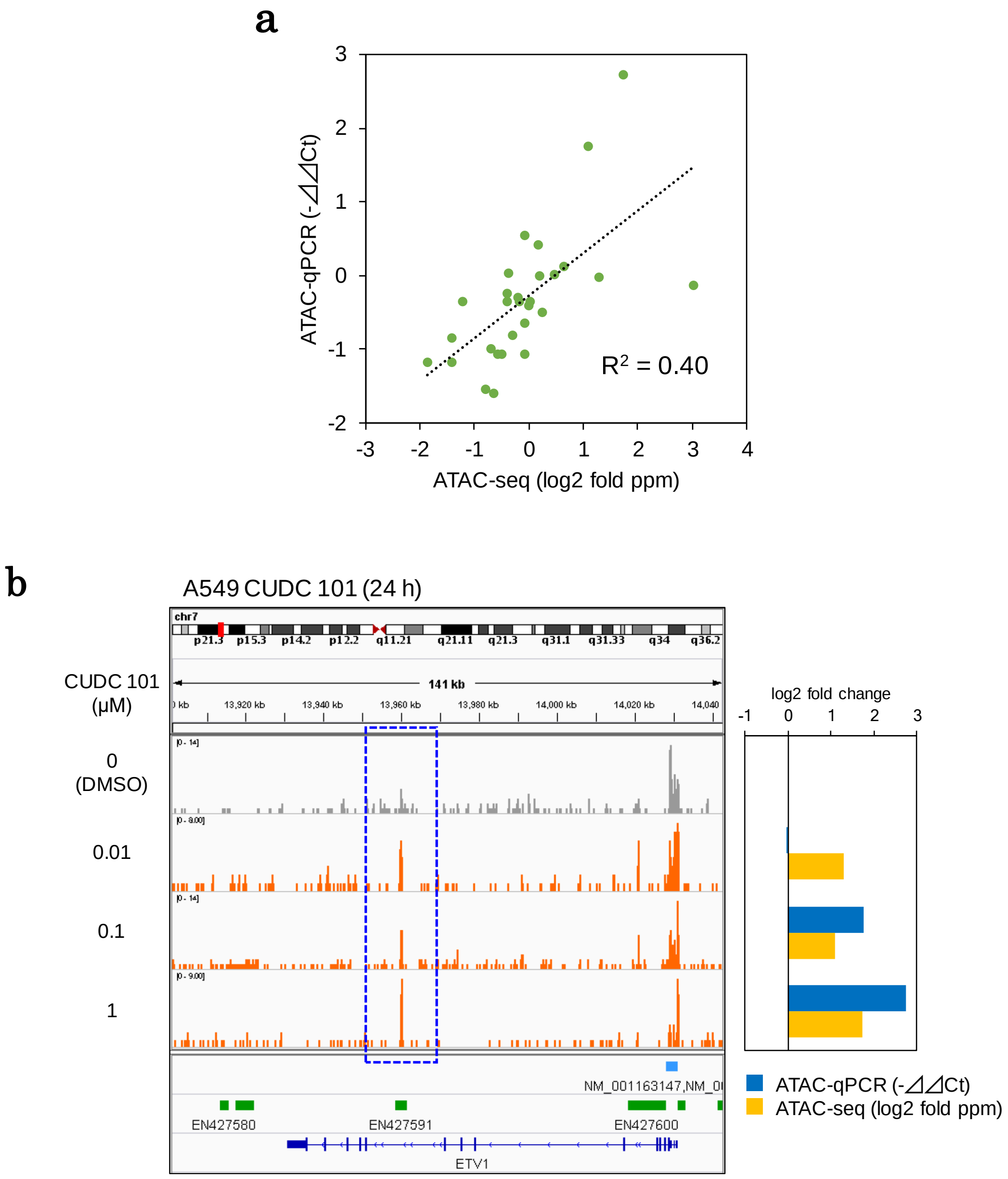

Supplementary Figure S6. qPCR validation of ATAC-seq.

(**a**) Fold changes in the chromatin accessibility in cells treated with CUDC 101 and (+)-JQ1 were compared between ATAC-seq and ATAC-qPCR. The Pearson correlation coefficient is shown in the inset. (**b**) Open chromatin status of A549 cells treated with CUDC 101 visualized in IGV (autoscale). Changes in chromatin accessibility, which are indicated in the dashed blue box, were evaluated by both ATAC-seq and ATAC-pPCR (the graph at right).

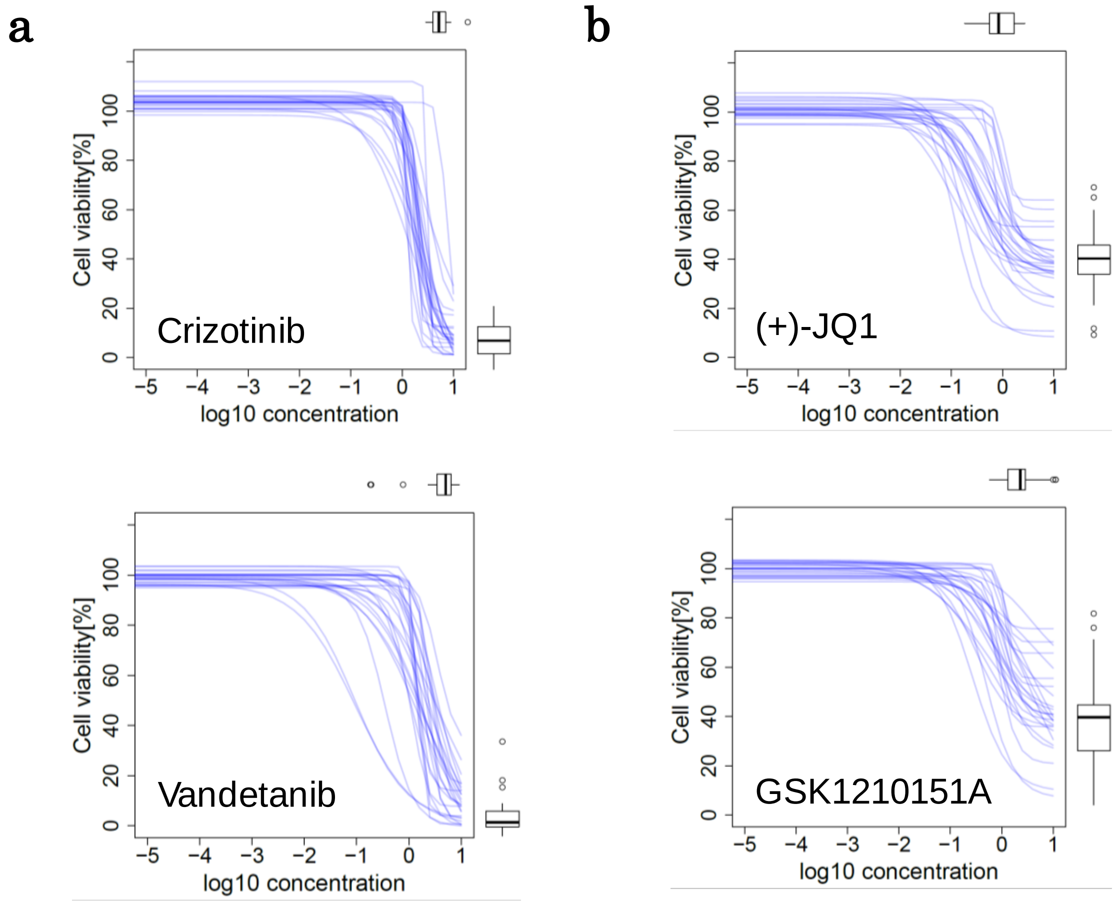

Supplementary Figure S7. The results of the cell viability test for representative cases.

The dose-response curves of 26 lung cancer cell lines are shown for two multi kinase inhibitors (**a**) and two epigenetic inhibitors (**b**). Each line indicates a cell line.

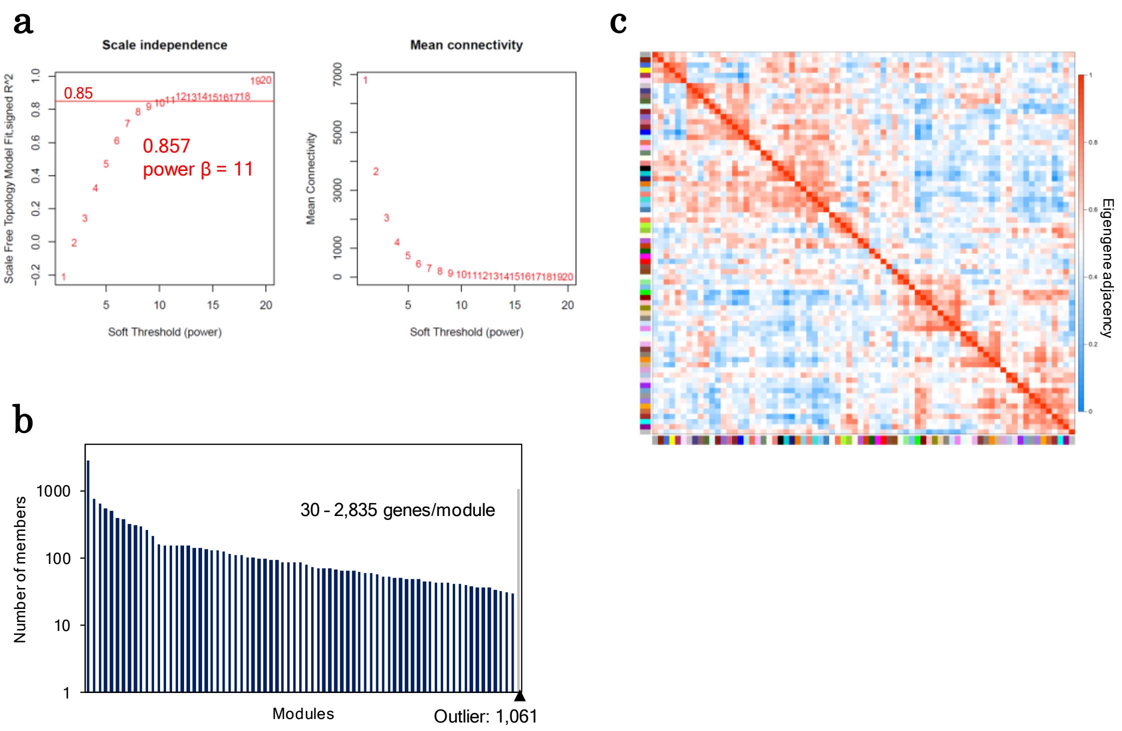

Supplementary Figure S8. WGCNA analysis.

(**a**) The soft-thresholding power of the network construction was determined using the WGCNA function. (**b**) The number of genes in each co-expression module. The modules were constructed by using from 30 to 2,835 genes, and 1,061 genes were classified as outliers. (**c**) The heat map of the adjacency of the module eigengenes. The modules were clustered according to the similarity of the module eigengenes. The colors represent each module.

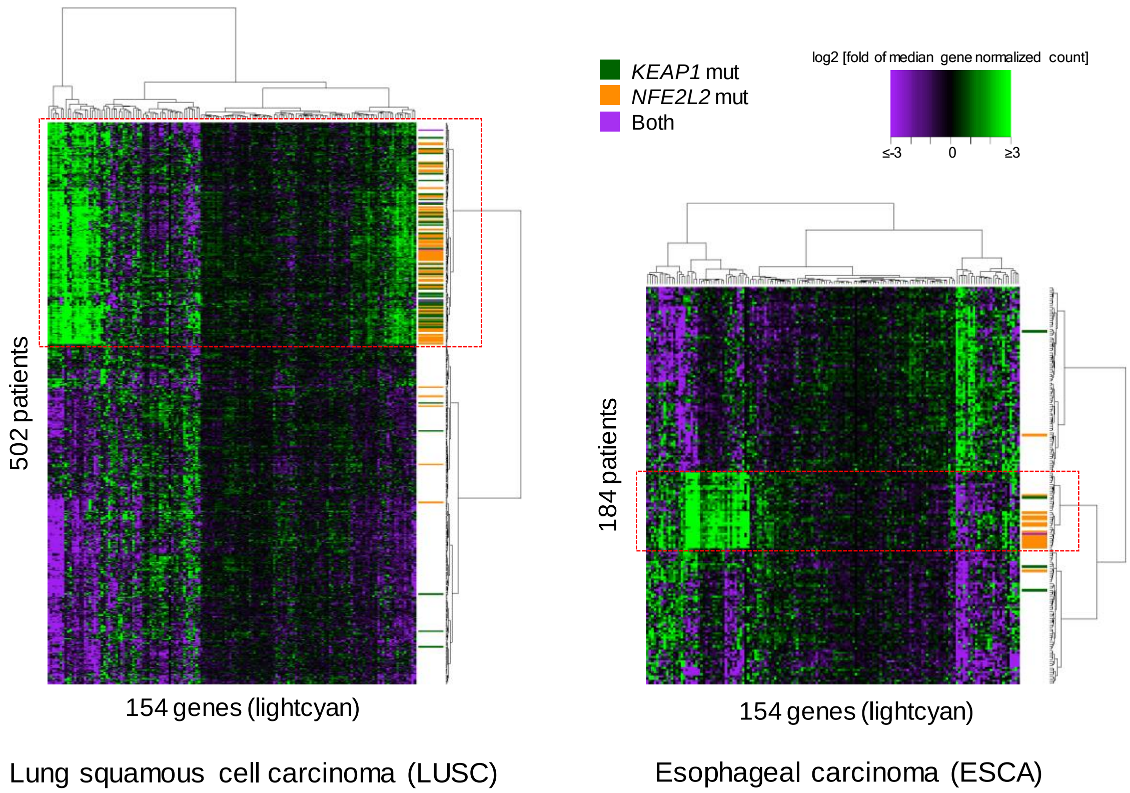

Supplementary Figure S9. Expression patterns of the "lightcyan" module and the *KEAP1-NFE2L2* mutational status according to the TCGA data.

Expression patterns of the "lightcyan" module members are shown in the TCGA-LUSC and TCGA-ESCA datasets. The color key is shown in the margin. The mutational status of *KEAP1* and *NFE2L2* are shown for each case, as indicated on the side of the heat map.

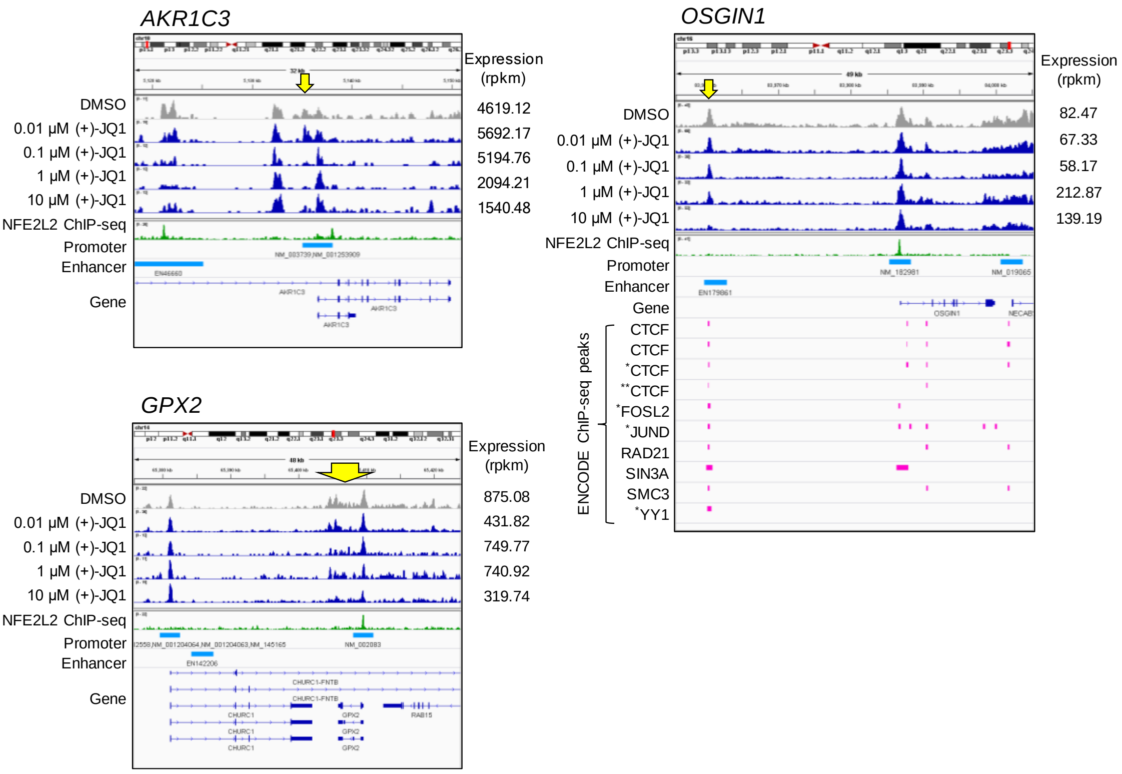

Supplementary Figure S10. Perturbation of chromatin accessibility by BET inhibitors.

Transcriptome and chromatin changes of selected "lightcyan" module members (A549, dataset 1, 24 h). The ChIP-seq data for NFE2L2 and other transcription factors were downloaded from the ENCODE website. *0.02% ethanol for 1 h; **100 nM dexamethasone for 1 h.

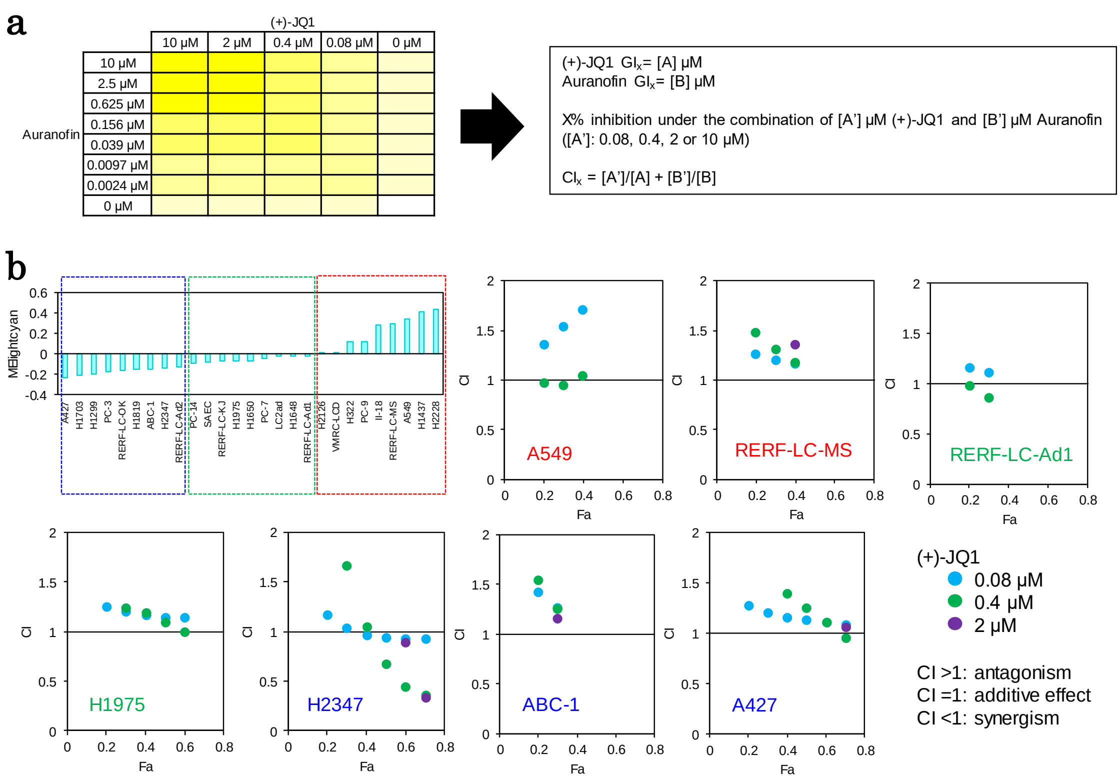

Supplementary Figure S11. Combination treatment with auranofin and (+)-JQ1.

(**a**) The design of the assay plate and the CI calculation for the combination treatment. The assay plate was designed to test eight conditions for auranofin and five conditions for (+)-JQ1 treatment. (**b**) The graph represents the activity of the "lightcyan" module in different cell lines. Cell lines with high, intermediate or low activity of the "lightcyan" module are indicated in red, green and blue, respectively. A CI-Fa plot is shown for eight cell lines. The color legend of the (+)-JQ1 conditions is shown in the margin.

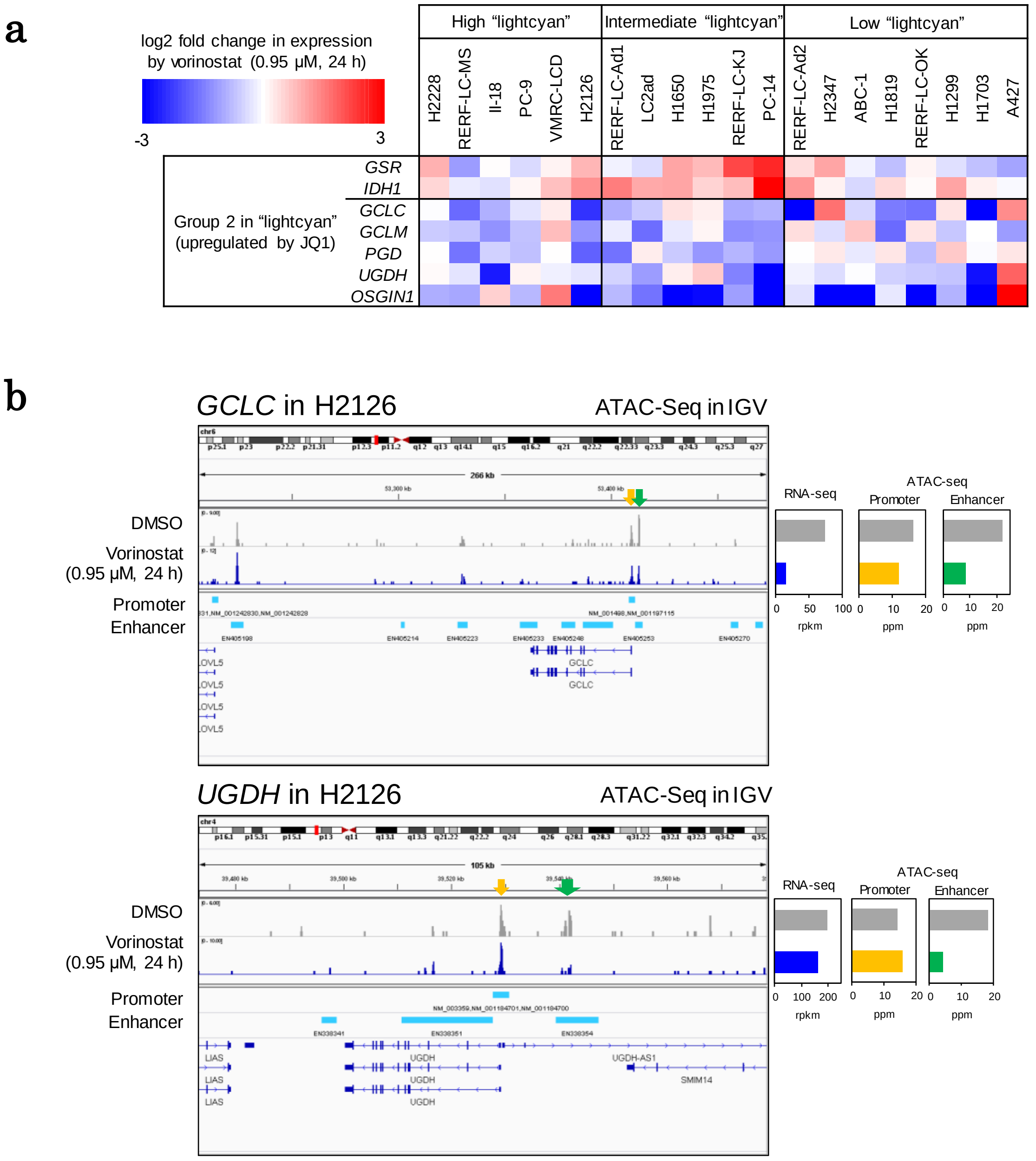

Supplementary Figure S12. Perturbation of expression and chromatin accessibility by vorinostat.

(**a**) The heat map shows the fold changes in the expression of group 2 genes during vorinostat treatment (dataset 2). The group 2 genes were partially downregulated by vorinostat. The color key is shown in the margin. (**b**) Open chromatin status of

GCLC (upper) and *UGDH* (lower) in H2126 cells treated with vorinostat (control: DMSO; dataset 2), as visualized by IGV (autoscale). The promoter and enhancer regions shown in this analysis are indicated by the yellow and green arrows, respectively. The graphs in the margin represent the abundances in expression (rpkm) and chromatin accessibility (ppm), as evaluated by RNA-seq and ATAC-seq.

References

1. Buenrostro, J. D., Giresi, P. G., Zaba, L. C., Chang, H. Y. & Greenleaf, W. J. Transposition of native chromatin for fast and sensitive epigenomic profiling of open chromatin, DNA-binding proteins and nucleosome position. *Nat. Methods* **10**, 1213–1218 (2013).
2. Milani, P. *et al.* Cell freezing protocol suitable for ATAC-Seq on motor neurons derived from human induced pluripotent stem cells. *Sci. Rep.* **6**, 25474 (2016).
3. Suzuki, A. *et al.* Aberrant transcriptional regulations in cancers: Genome, transcriptome and epigenome analysis of lung adenocarcinoma cell lines. *Nucleic Acids Res.* **42**, 13557–13572 (2014).
4. Langfelder, P. & Horvath, S. WGCNA: an R package for weighted correlation network analysis. *BMC Bioinformatics* **9**, 559 (2008).
5. Dobin, A. *et al.* STAR: Ultrafast universal RNA-seq aligner. *Bioinformatics* **29**, 15–21 (2013).
6. Langmead, B. & Salzberg, S. L. Fast gapped-read alignment with Bowtie 2. *Nat. Methods* **9**, 357–9 (2012).
